# Supplementary material for: Confidence-guided cryo-EM map optimisation with LocScale-2.0
Source: Nat Commun. 2026 Jul 17;17:8778. doi: 10.1038/s41467-026-75327-8 (PMC13493958; doi:10.1038/s41467-026-75327-8)
Supplement: Supplementary file 1 [file 41467_2026_75327_MOESM1_ESM.pdf]

Supplementary Information

Confidence-guided cryo-EM map optimisation with LocScale-2.0

Alok Bharadwaj[a], Reinier de Bruin[a], and Arjen J. Jakobi[a]

[a] Department of Bionanoscience, Kavli Institute of Nanoscience, Delft University of Technology, 2629HZ, The Netherlands

Supplementary Figures

1 **Definition of pseudoatom placement and generation of hybrid models** . . . 2
2 **Pseudomodel generation and ADP refinement** . . . 3
3 **Implementation and validation of pseudomodel ADP refinement** . . . 4
4 **LocScale-2.0 map optimisation** . . . 5
5 **Automated model building with LocScale-2.0 maps** . . . 6
6 **Monte Carlo Dropout and pVDDT scores** . . . 8
7 **Interpretation of LocScale-FEM maps** . . . 9
8 **LocScale-FEM interpretation of small-molecule ligands** . . . 10
9 **Output structure of LocScale-2.0** . . . 11

Supplementary Tables

1 Computation time for Hybrid LocScale-2.0 and LocScale-FEM optimisation . . . 12
2 Scaling of LocScale-FEM compute time with volume. . . . 12
3 EMDB and PDB IDs for maps and models used for MC-EMmerNet training . . . 13
4 EMDB and PDB IDs for maps and models used for MC-EMmerNet validation . . . 14
5 EMDB and PDB IDs for maps and models used for testing and uncertainty calibration . . . 14
6 EMDB and PDB IDs for maps and models in additional test dataset . . . 14
7 Details of MC-EMmerNet architecture . . . 15

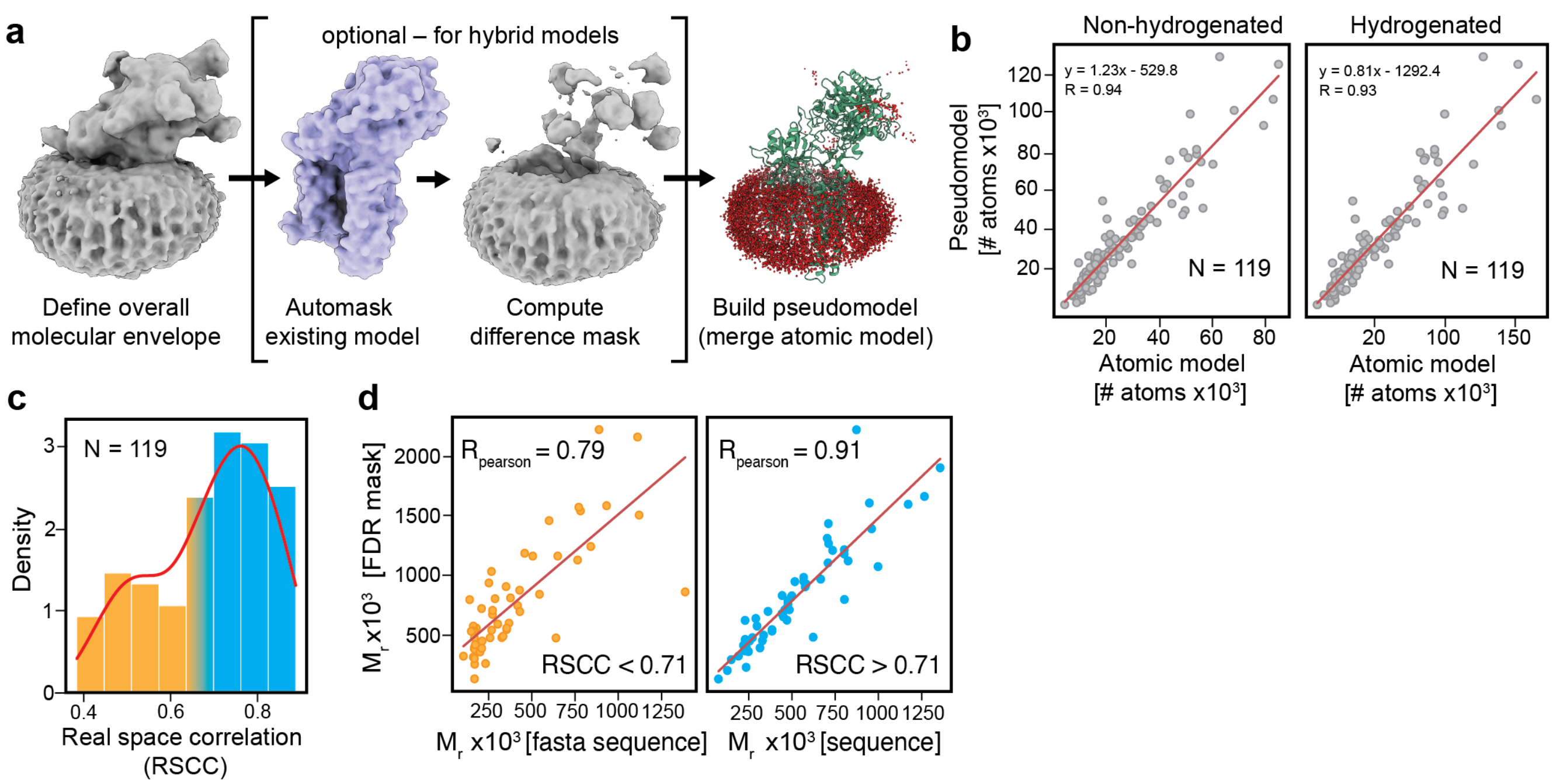

Supplementary Fig. 1. | **Definition of pseudoatom placement and generation of hybrid models.** (a) Illustration of pseudoatom placement for human γ-secretase (EMDB: EMD-3061). The molecular envelope is estimated using FDR thresholding[52], defined as the volume enclosed by a binarised mask computed from the confidence map at a 1% FDR threshold. When (partial) prior model information is available, a contextual structure mask is derived from the symmetric difference between the binarised model and envelope masks. Pseudoatoms are placed only within this difference mask and then merged with the prior atomic model. (b) Regression plots showing the number of pseudoatoms placed within the atomic model mask versus the expected number of atoms based on the known protein sequence, assuming hydrogenated and non-hydrogenated models. (c) Histogram of real-space correlation coefficients (RSCC) between FDR confidence masks and atomic model masks. Bars are colored by RSCC values below (RSCC < 0.71, orange) and above (RSCC > 0.71, blue) the median. (N = 119 for panels b–c.) (d) Regression plots comparing molecular mass estimates derived from pseudoatom placements within the FDR mask to those from the primary sequence, for RSCC below and above the median. Source data are provided as a Source Data file.

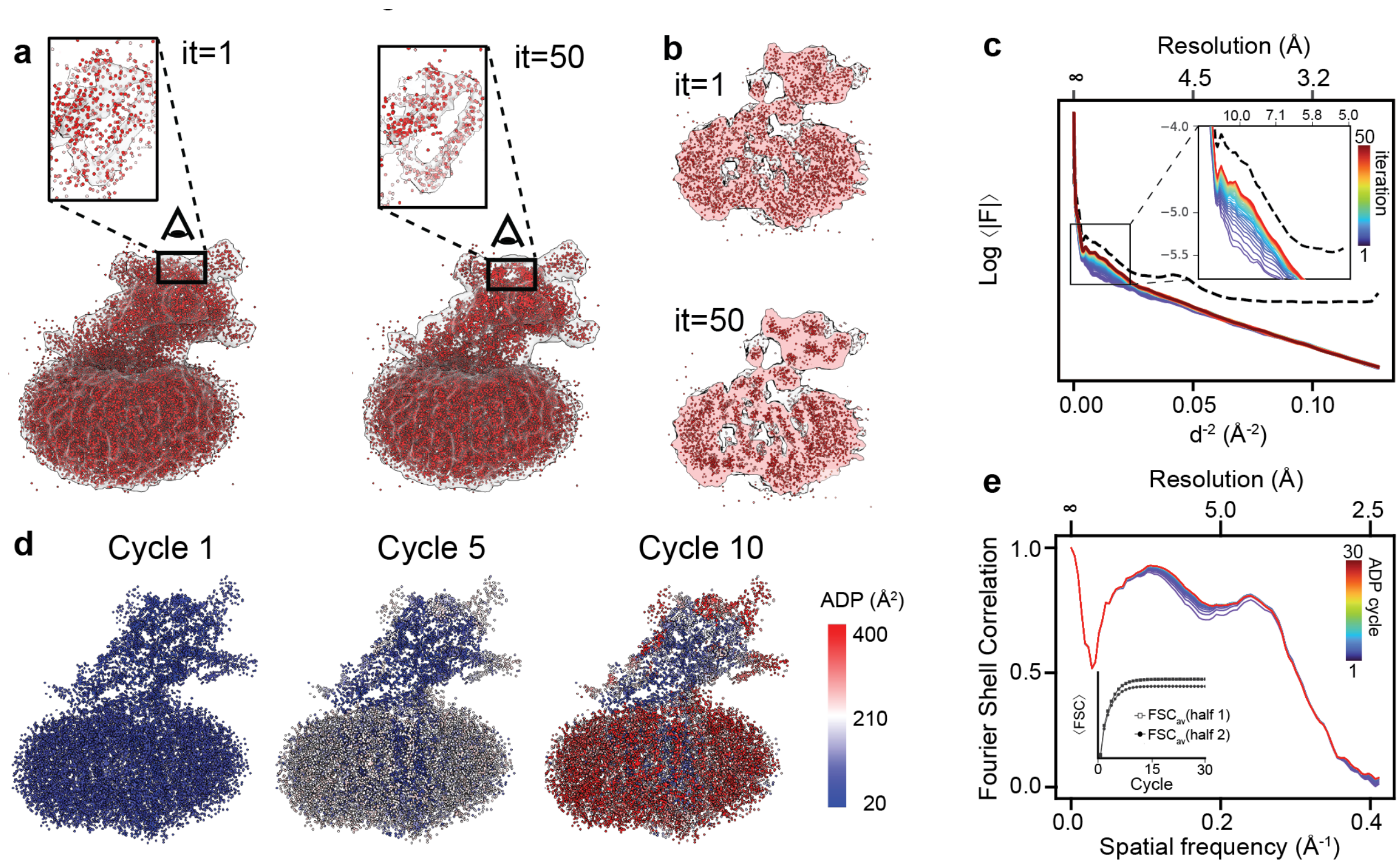

Supplementary Fig. 2. | **Pseudomodel generation and ADP refinement.** (a) Example for pseudoatom placement and refinement for human γ-secretase (EMDB: EMD-3061). Models are shown for the start (iteration 1) and end (iteration 50) of the positional refinement. The molecular boundary is outlined by a 1% FDR confidence mask. (b) Slab through the pseudomodel for both refinement iterations. (c) Radially averaged Fourier amplitudes of model maps computed throughout the iterations of pseudomodel refinement. Inset: Close-up of low-to-medium resolution region defining the overall molecular envelope. (d) Atomic displacement factors (ADP) mapped onto atomic coordinates shown for different snapshots across the ADP refinement cycles. (e) Evolution of model-to-map Fourier shell correlation (FSC) shown for a series of ADP refinement cycles. The insets shows the half-map validation displaying the average integrated FSC for work and test maps as a function of refinement cycle. Source data are provided as a Source Data file.

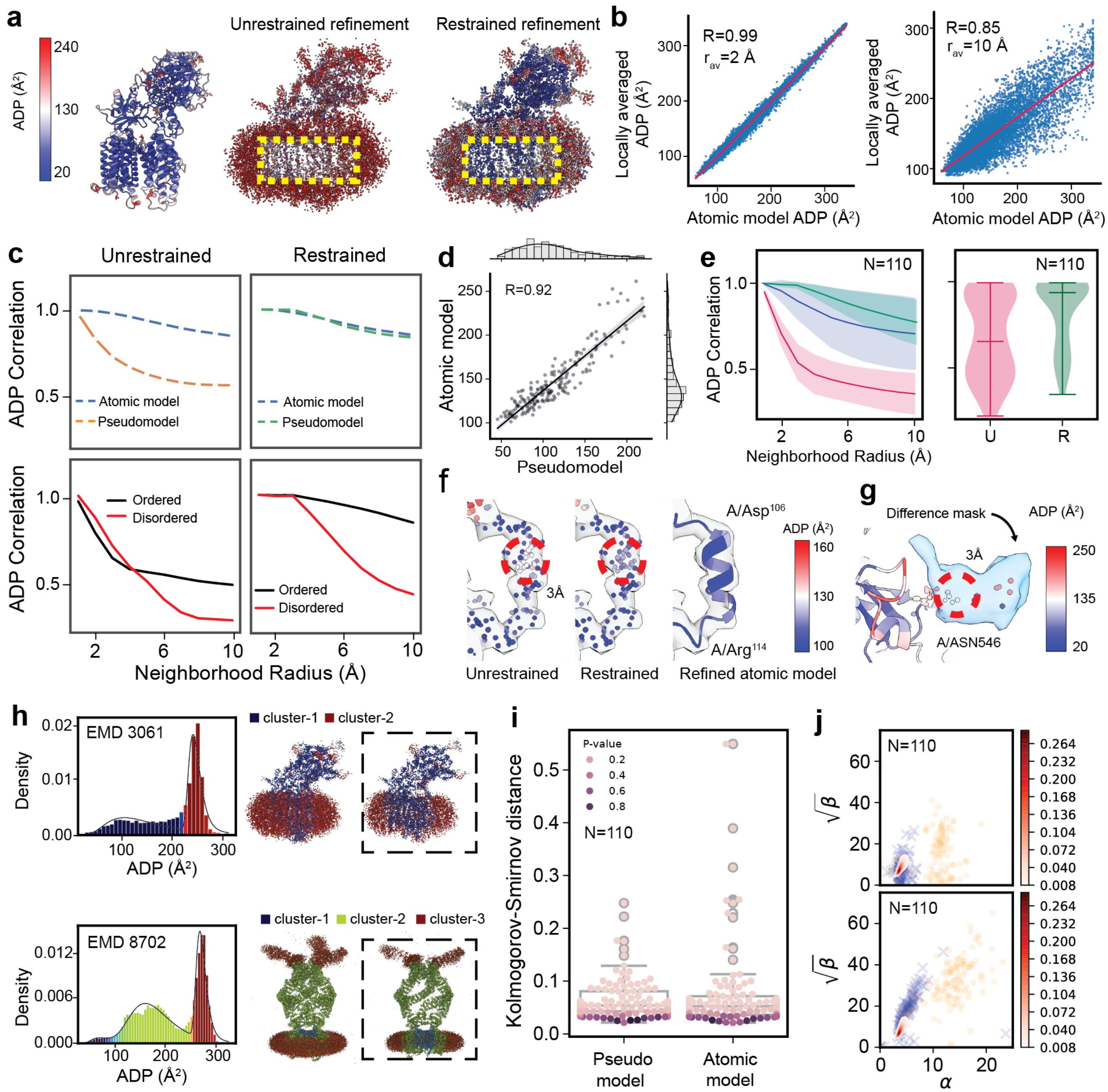

Supplementary Fig. 3. | **Implementation and validation of pseudomodel ADP refinement.** (a) ADP distribution mapped on the refined atomic model of human gamma secretase complex (PDB ID 5a63). (b) Scatter plot of atomic ADPs versus locally averaged pseudomodel ADPs for averaging radii of 2 Å (left) and 10 Å (right). (c) Relationship between neighborhood radius and ADP correlation coefficient for unrestrained and restrained ADP refinement of pseudomodels compared to atomic model refinement (top). Also shown are neighborhood radius correlation curves for pseudoatom clusters in ordered and disordered regions of the map. (d) Regression plot of atomic model vs. pseudomodel ADPs for γ-secretase (EMD-3061/PDB ID 5a63). (e) Left: ADP correlation as a function of neighbourhood radius plotted for 110 randomly selected map/model pairs from the EMDB (red: unrestrained pseudomodel ADP refinement; green: restrained pseudomodel ADP refinement; blue: atomic model ADPs). Right: Similarity of the ADP correlation curves to a reference curve derived from an atomic model. (f) Effect of neighbourhood-restrained ADP refinement of pseudoatoms shown after four ADP refinement cycles. Red circles outline the 3 Å averaging radius (left and middle). For comparison refined ADPs of an explicit atomic model (PDB ID 5a63) are also shown for the same map segment (right). (g) ADP restraints at the boundary of explicitly modelled and pseudoatom regions in hybrid reference models. The difference mask outlines the unmodelled density of a N-linked glycan. (h) ADP validation with toBvalid[39] for pseudomodels obtained for EMD-3061 and EMD-8702. Histograms shows distinguishable modes of the shifted inverse Γ distributions (EMD-3061: two modes; EMDB: EMD-8702: three modes). Mapping the different modes back onto the pseudomodel reveals dinstinguishable clusters corresponding to well-resolved and poorly resolved/disordered regions (right). (i) Plot of Kolmogorov-Smirnov distances measuring the maximum distance between the empirical cumulative distribution function of the pseudoatom or atomic model ADP distributions and the cumulative distribution function of the theoretical gamma distribution for 110 randomly selected maps/models from the EMDB. Statistical significance is obtained from permutation tests with 1000 resamples. (j) α versus $\beta^{1/2}$ plots for bimodal fits to ADP distributions from atomic models (top) and pseudomodels (bottom). The red comet shows the smoothened unimodal distribution of α versus $\beta^{1/2}$ from empirical analysis of 45,000 PDB structures[39]. Source data are provided as a Source Data file.

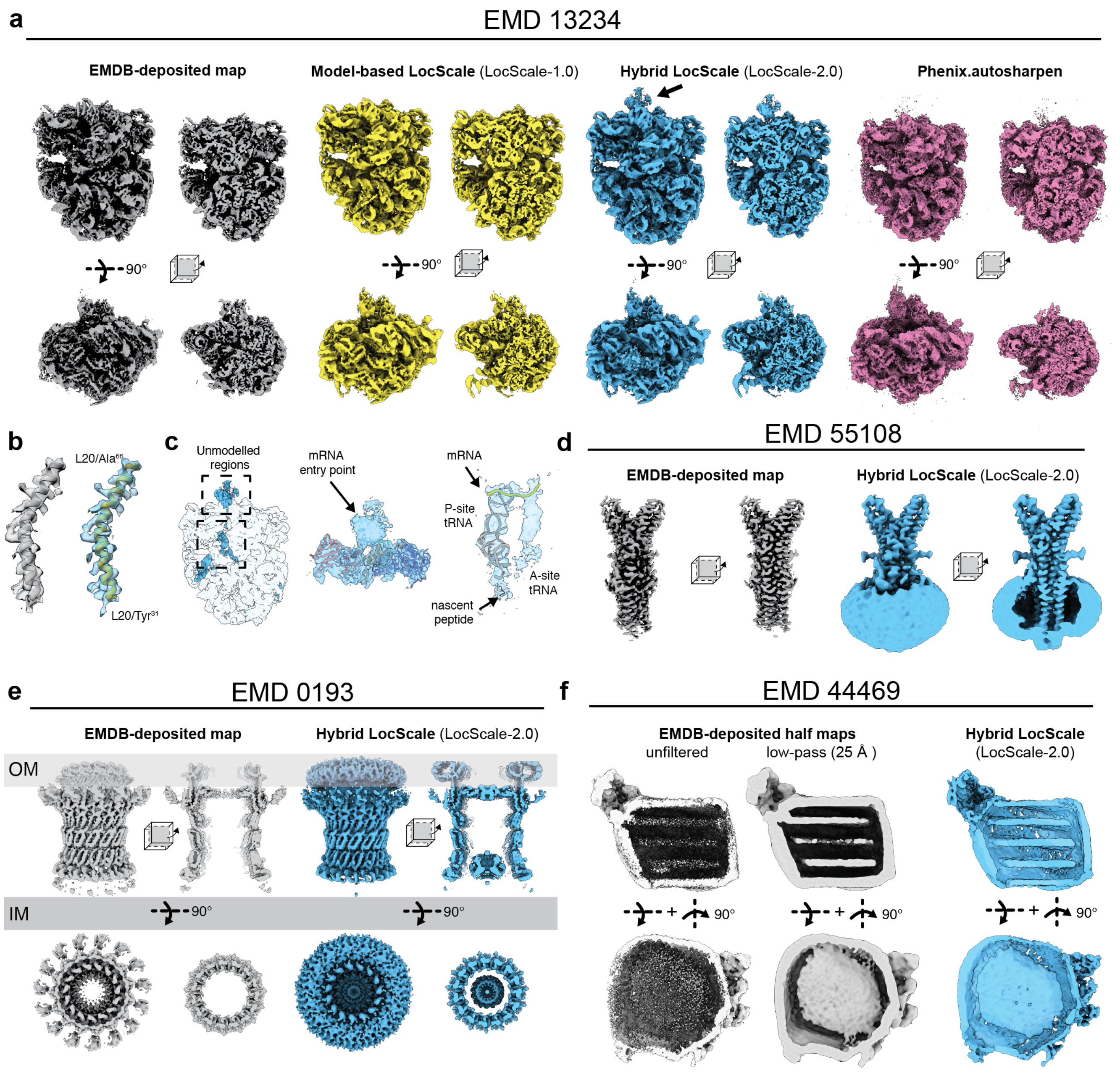

Supplementary Fig. 4. | **LocScale-2.0 map optimisation.** (a) On-views and cross-sectional views of locally sharpened maps for in situ subtomogram averages of *Mycoplasma pneumoniae* 70S ribosomes (EMDB: EMD-13234) using model-based sharpening with LocScale-1.0 (middle left), hybrid reference sharpening in LocScale-2.0 (middle right) and phenix.autosharpen (right). The author-deposited map is also shown (left). Arrows mark the mRNA entry point. (b) Side-by-side comparison of author-deposited and LocScale-2.0 maps for a well-resolved helical density segment from (a). (c) Unmodelled and partially interpretable regions of contextual structure highlighted in the LocScale-2.0 map. (d) Comparison of author-deposited and LocScale-2.0 sharpened maps for *Klebsiella pneumoniae* type II secretion system outer membrane complex (EMD-0193). (e) Comparison of author-deposited and LocScale-2.0 sharpened maps for Tweety homologue 2 (TTYH2, EMDB: EMD-51108). (f) Unfiltered and low-pass filtered half maps in comparison to the hybrid LocScale map for ApoB100 (EMDB: EMD-44469).

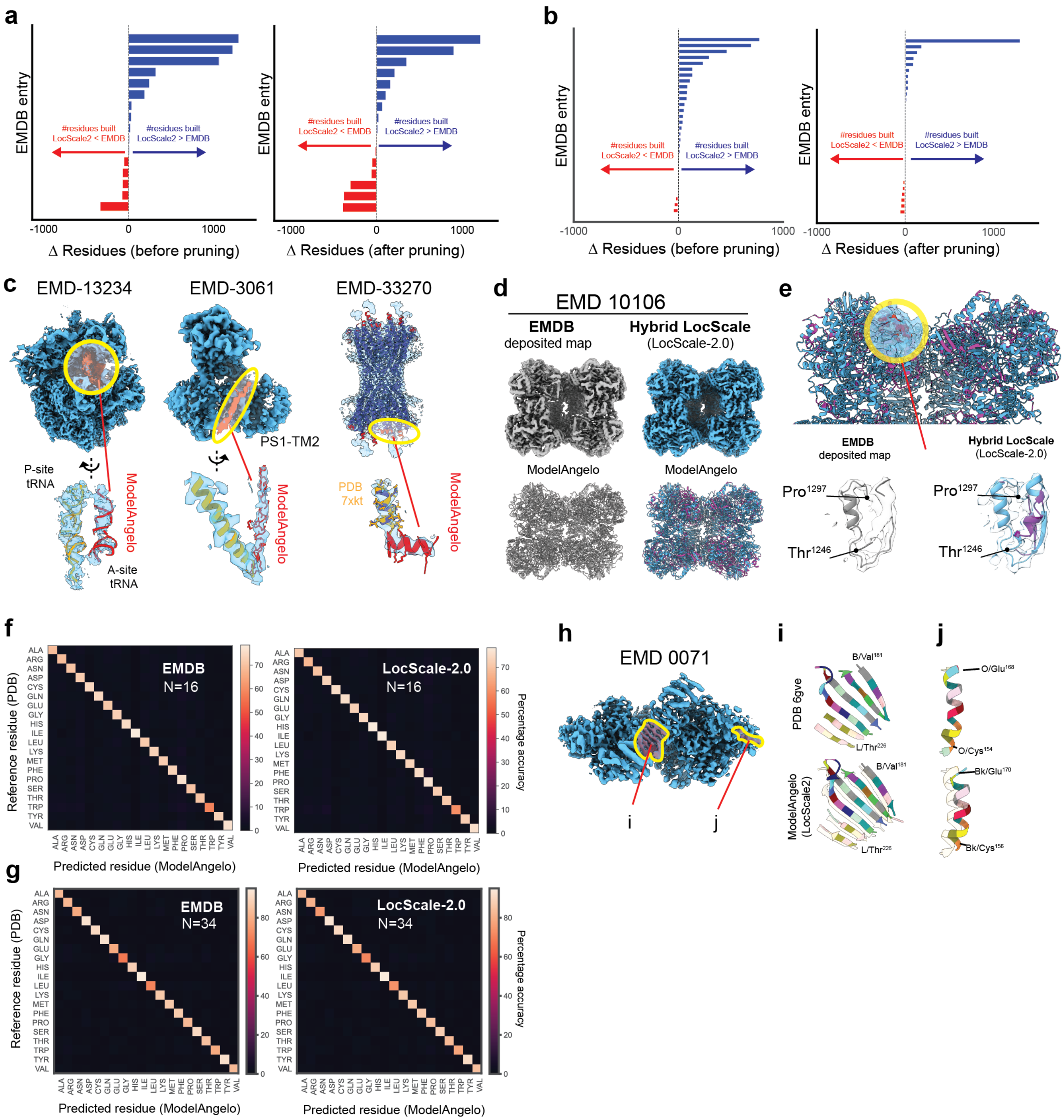

Supplementary Fig. 5. | **Automated model building with LocScale-2.0 maps.** (a,b) Aggregate statistics of the number of ModelAngelo-built residues for 50 randomly selected maps from the EMDB before and after pruning; residue numbers are not normalised for point group symmetry. 16 of these models (a) were not part of the Modelangelo training set, and 34 had been used in ModelAngelo training (b). (c) Selection of EMDB entries for which LocScale-2.0 maps support improvements in ModelAngelo model completion compared to the deposited maps. Residues and nucleotide bases additionally built in LocScale-2.0 maps are shown in red. (d) LocScale-2.0 map for *Azospirillum brasilense* glutamate synthase (EMDB: EMD-10106). (e) Automatically built models from ModelAngelo superposed on a selected map region indicated in (a) using the author-deposited map (left) and LocScale-2.0 map (right) as input. The full map was used for automated model building. (f) Sequence recall for ModelAngelo predictions using deposited or LocScale-2.0 maps for 16 models not part of the ModelAngelo training dataset. (g) Sequence recall for ModelAngelo predictions using deposited or LocScale-2.0 maps for 34 models that had been used in the ModelAngelo training dataset. (h-j) Sequence recall visualised in EMDB: EMD-0071/PDB: 6gve. Two representative regions are shown, one of which (j) shows a coordinate shift in the LocScale-2.0 map. Source data are provided as a Source Data file.

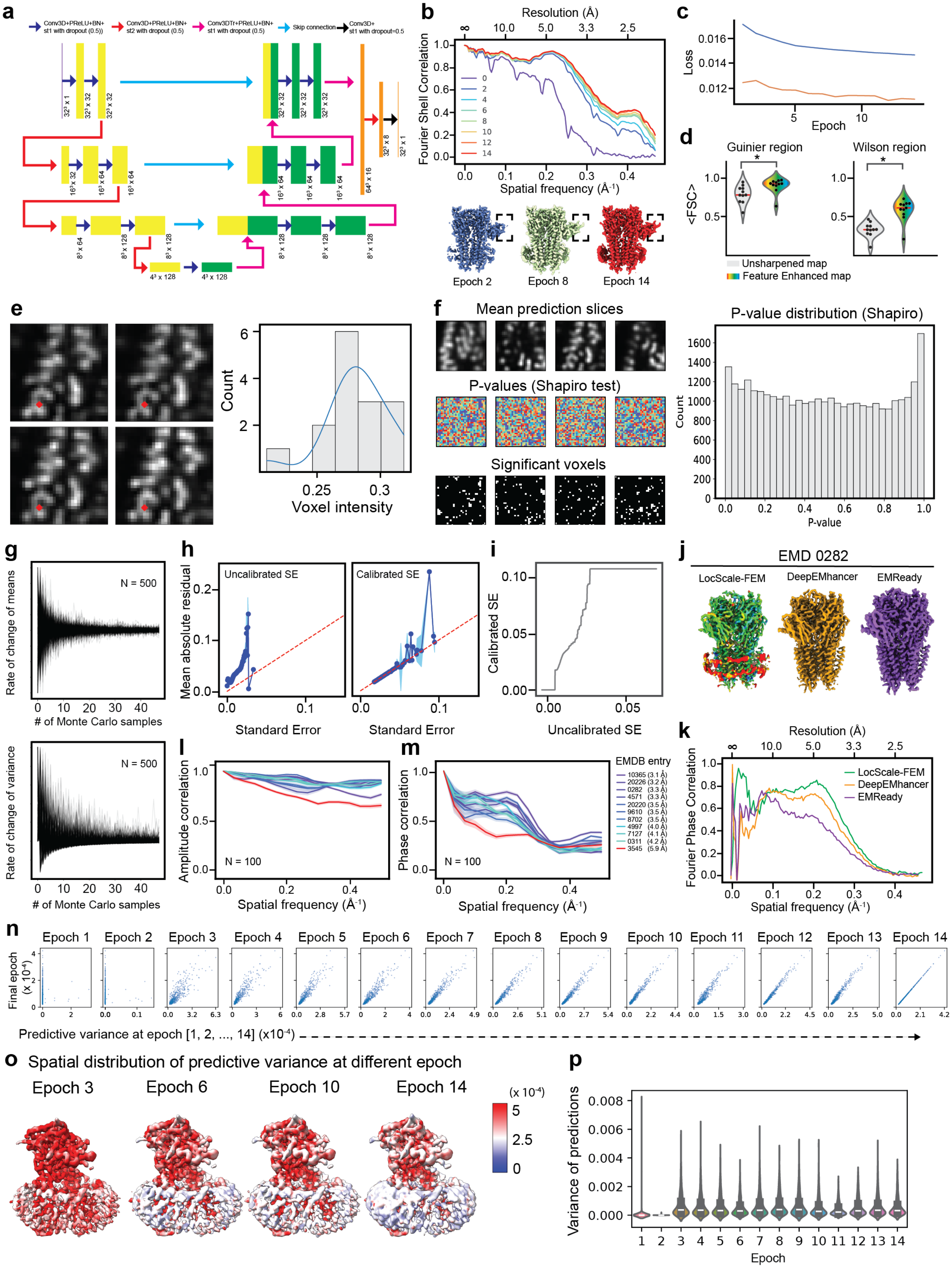

a
Conv3D+PReLU+BN+ st1 with dropout (0.5))
Conv3D+PReLU+BN+ st2 with dropout (0.5)
Conv3DTr+PReLU+BN+ st1 with dropout (0.5)
Skip connection
Conv3D+ st1 with dropout=0.5
b
Resolution (Å)
Fourier Shell Correlation
Spatial frequency (Å-1)
Epoch 2
Epoch 8
Epoch 14
c
Loss
Epoch
d
Guinier region
Wilson region
<FSC>
Unsharpened map
Feature Enhanced map
e
Count
Voxel intensity
f
Mean prediction slices
P-values (Shapiro test)
Significant voxels
P-value distribution (Shapiro)
Count
P-value
g
Rate of change of means
N = 500
# of Monte Carlo samples
Rate of change of variance
h
Mean absolute residual
Uncalibrated SE
Calibrated SE
Standard Error
i
Calibrated SE
Uncalibrated SE
j
EMD 0282
LocScale-FEM
DeepEMhancer
EMReady
k
Resolution (Å)
Fourier Phase Correlation
Spatial frequency (Å-1)
l
Amplitude correlation
N = 100
m
Phase correlation
EMDB entry
n
Final epoch (x 10-4)
Predictive variance at epoch [1, 2, ..., 14] (x10-4)
o
Spatial distribution of predictive variance at different epoch
Epoch 3
Epoch 6
Epoch 10
Epoch 14
p
Variance of predictions
Epoch

Supplementary Fig. 6. | **Monte Carlo Dropout and pVDDT scores.** (a) 3D U-Net architecture of MC-EMmerNet. b) Spectral behaviour of MC-EMmerNet training measured by Fourier-Shell Correlation (FSC) of MC-EMmerNets test set prediction vs. target map as a function of training epoch. Low frequency information is rapidly learnt after a few epochs, while high frequency detail is learnt more gradually as observed by the slower convergence in the FSC curves at high frequencies. Predicted maps at selected epochs are shown. (c) Loss curves curves showing the evolution of training and validation losses. Due to the presence of dropouts in training the validation loss is lower than the training loss. (d) Average Fourier Shell correlation (<FSC>) in the Guinier region (0-0.1 $Å^{-1}$) and Wilson region (>0.1 $Å^{-1}$) measured vs. target map for the entire test set (N=11). (e) Central slices from 4 out of 15 samples generated by the MC-EMmerNet model using Monte Carlo dropout. The intensity distribution for the voxel highlighted in red is shown on the right. (f) Normality tests for all voxels in the sample cubes, with a histogram of the resulting p-values shown to the right. (g) Effect of the number of Monte Carlo samples on the change in prediction mean (top) and variance (bottom). (h) Reliability and calibration curves used to evaluate the uncertainty calibration. (i) Calibration curve from isotonic regression. (j) Feature-enhanced map (LocScale-FEM) for EMD-0282 colored by pVDDT scores, alongside DeepEMhancer and EMReady predictions. (k) Fourier phase correlation between the three deep learning models shown in (h) and the unmodified reference map. (l) Fourier amplitude correlation between predicted and target (hybrid LocScale-2.0) map for 11 EMDB entries with varying resolution between 3-6 Å. Shown are the mean correlations between 100 randomly selected signal cubes with their 95% confidence intervals. (m) Fourier phase correlation between predicted and unmodified (average of EMDB-deposited half maps) for 11 EMDB entries with varying resolution between 3-6 Å. Shown are the mean correlations between 100 randomly selected signal cubes with their 95% confidence intervals. (n) Scatter plots comparing voxel-wise predictive variance estimated at different training epochs with the variance obtained after the final epoch (epoch 15). Each panel shows 5,000 randomly sampled voxels. Rapid convergence toward the final variance estimates is observed after the early epochs. (p) Spatial distribution of predictive variance mapped onto the contoured density of EMD-3061 at selected epochs. Variance patterns stabilise early during training. (o) Distribution of predictive variance across epochs shown as violin plots. The distributions remain consistently right-skewed with only a small fraction of high-variance voxels, while the bulk of the distribution stabilises after approximately the third epoch, indicating convergence of the posterior predictive variance. Source data are provided as a Source Data file.

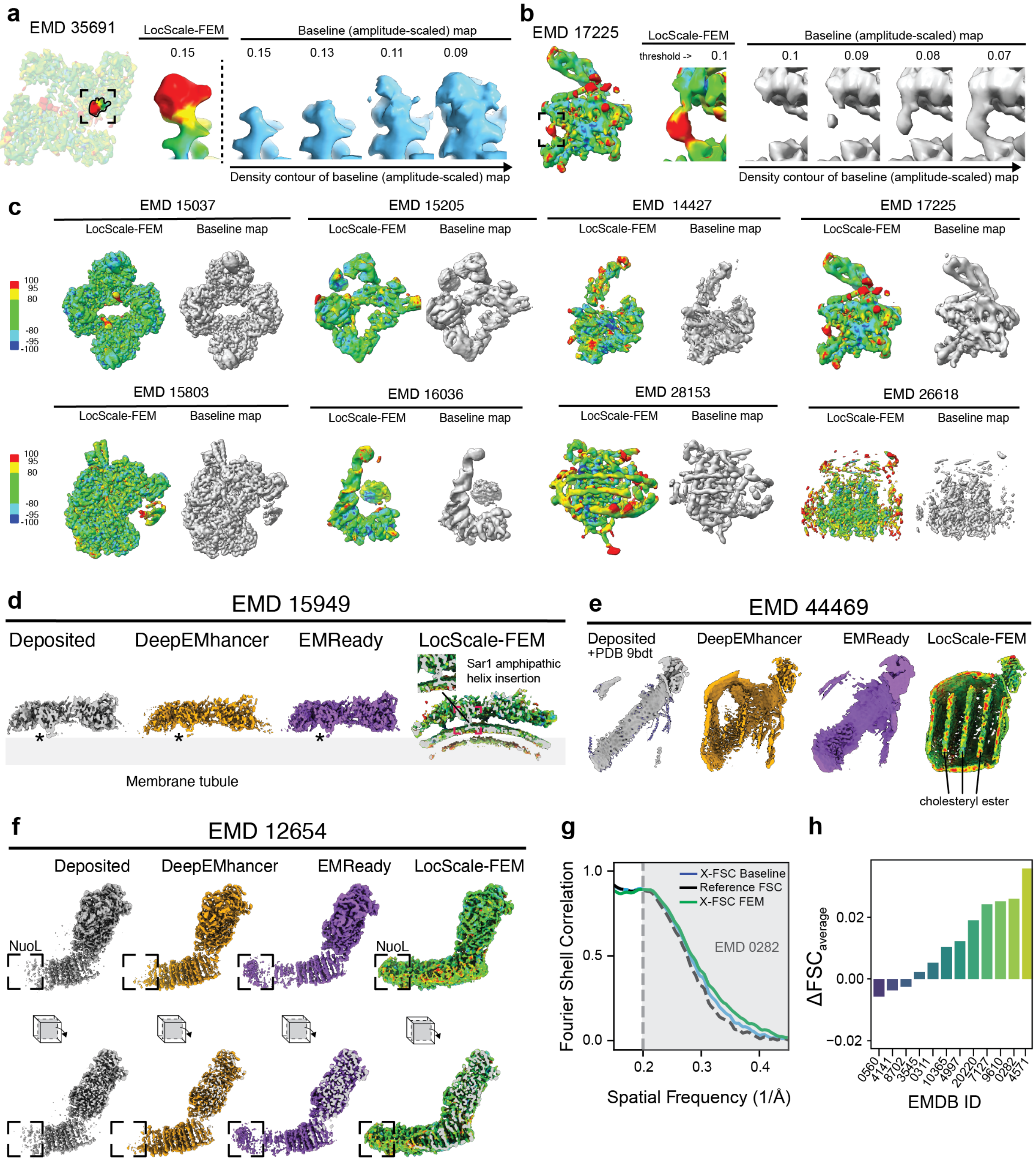

Supplementary Fig. 7. | **Interpretation of LocScale-FEM maps.** (a) LocScale-FEM map for EMDB: EMD-35691) shown at 0.15 contour level. A feature with strong positive pVDDT score is shown as close-up. The corresponding baseline map of the highlighted feature displayed at successively decreasing contour levels is also shown. (b) LocScale-FEM map for EMDB: EMD-17225 shown at 0.15 contour level. A feature with strong positive pVDDT score is shown as close-up. The corresponding baseline map of the highlighted feature displayed at successively decreasing contour levels is also shown. (c) LocScale-FEM maps for eigth random EMDB entries shown alingsude their baseline maps. The maps are sorted according to increasing variance in the pVDDT scores. (d) Comparison of optimised maps for subtomogram averages of the yeast COPII inner coat assembled on membrane tubules (EMDB: EMD-15949). The inset shows density for the membrane-embedded amphipathic helix of the Sar1 subunit in the LocScale-FEM map. (e) Comparison of maps for Apolipoprotein B-100/low-density lipoprotein (LDL) particles EMDB: EMD-44469). Contextual densities for liquid-crystalline cholesteryl esters are highlighted. (f) On-axis and cross-sectional views of author-deposited and optimised maps for *E. coli* respiratory complex I (EMDB: EMD-12654. The peripheral antiporter subunit NuoL is highlighted. (g) Representative cross-FSC curves for EMD-0282 comparing baseline (blue) and LocScale-FEM (green) maps against the independent half-map reference (black). LocScale-FEM preserves or improves cross-half map agreement across spatial frequencies. (h) Average difference in cross-$FSC_{average}$ ($\Delta FSC_{average}$) at spatial frequencies beyond 0.2 $Å^{-1}$ across a representative set of EMDB entries. Predominantly positive values indicate that feature-enhanced maps improve agreement with independent data. Source data are provided as a Source Data file.

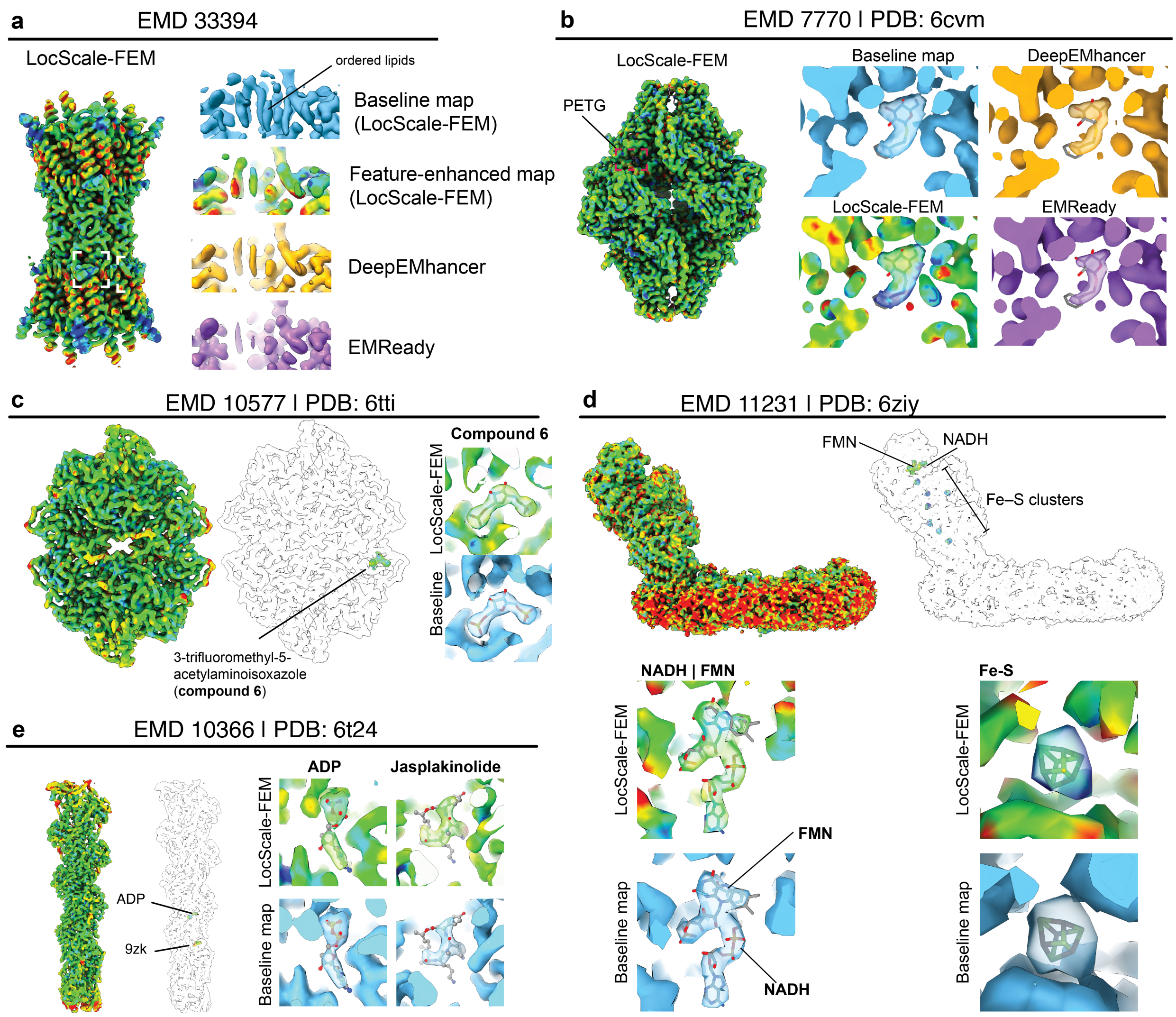

Supplementary Fig. 8. | **LocScale-FEM interpretation of small-molecule ligands.** (a) Suppression of ordered lipid densities in Connexin 43 (EMDB: EMD-33394) in LocScale-FEM, DeepEMhancer and EMReady maps displayed at equivalent normalised thresholds. (b) Phenylethyl-β-D-thiogalactopyranoside (PETG) binding site in β-galactosidase (EMDB: EMD-7770) in LocScale-FEM, DeepEMhancer and EMReady maps displayed at equivalent normalised thresholds. PETG is shown in stick representation. (c) LocScale-FEM map of fragment-complexed human pyruvate kinase PKM (EMDB: EMD-10577). Close-ups show LocScale-FEM feature-enhanced and baseline maps of the ligand binding site. (d) LocScale-FEM map of fragment-complexed *T. thermophilus* complex I (EMDB: EMD-11231). Close-ups show LocScale-FEM feature-enhanced and baseline maps of the flavin mononucleotide/NADH site and an F_4S_4 iron-sulfur cluster, respectively. (e) LocScale-FEM map of jasplakinolide-stabilised F-actin (EMDB: EMD-10366). Close-ups show LocScale-FEM feature-enhanced and baseline maps of the ADP site and an jasplakinolide binding sites, respectively.

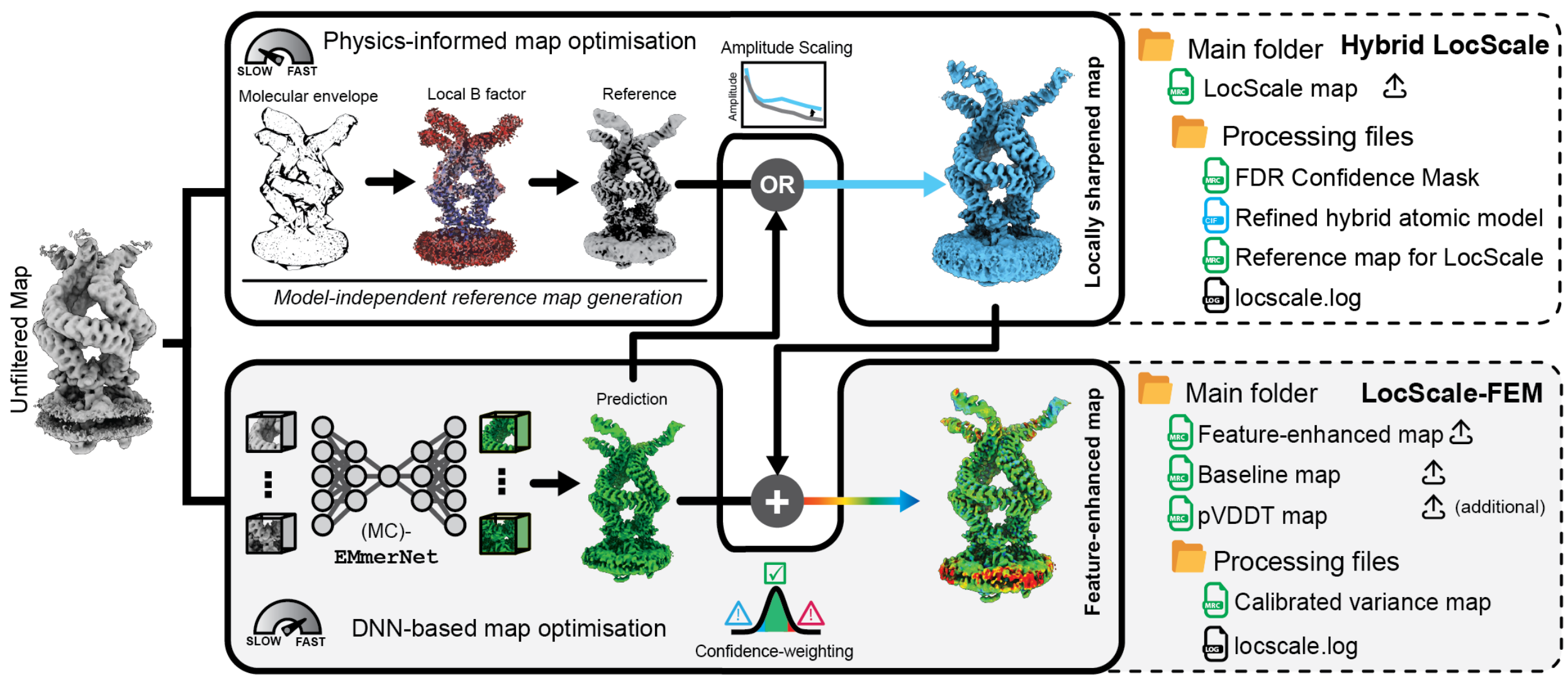

Supplementary Fig. 9. | **Output structure of LocScale-2.0.** Overview of the main output files generated by the Hybrid LocScale and LocScale-FEM workflows, including final optimised maps and intermediate processing files used for map optimisation and uncertainty estimation.

Supplementary Table 1. Computation time for Hybrid LocScale-2.0 and LocScale-FEM optimisation

	**EMD-3061**	**EMD-8702**	**EMD-44469**
Shape	180×180×180	400×400×400	336×336×336
1% FDR volume [Å^3]	2.5×10^5	1.9×10^6	5.3×10^6
**Hybrid LocScale**			
Number of pseudoatoms	9284	30887	206162
Number of atoms	10003	41900	38899
Pseudo-model generation [min]	∼4	∼18	∼105
ADP refinement [min]	∼14	∼72	∼117
**LocScale-FEM**			
Prediction [min]§	∼3	∼10	∼14
**Summary**			
Factor speed-up	6	9	16

§ LocScale-FEM predictions were done on a single Titan X (Pascal) GPU with 12 GB memory

Supplementary Table 2. Scaling of LocScale-FEM compute time with volume.

EMDB / N	Box size (voxels)	Unit cell (Å)	Volume (×10^6 Å^3)	Compute time (s)	Notes
4997	232	249.4	15.5	∼280	example
7127	256	335.3	37.7	∼530	example
8702	400	486.2	114.9	∼1200	example
3545	360	572.4	187.5	∼4000	example
N=4	–	200–250	8–15	350 ± 200	mean ± s.d.
N=7	–	250–400	15–64	600 ± 300	mean ± s.d.
N=3	–	400–600	64–216	2600 ± 1150	mean ± s.d.

Supplementary Table 3. EMDB and PDB IDs for maps and models used for MC-EMmerNet training

#	EMDB	PDB	#	EMDB	PDB	#	EMDB	PDB
1	0026	6gl7	2	0038	6gml	3	0071	6gve
4	0093	6gyn	5	0094	6gyo	6	0132	6h3c
7	0408	6nbd	8	0415	6nbq	9	0452	6nmi
10	0490	6nr8	11	0492	6nra	12	0589	–
13	0592	6o1m	14	0776	6ku9	15	10049	6rx4
16	10069	6s01	17	10100	6s5t	18	10106	6s6u
19	10273	6sof	20	10279	6sp2	21	10324	6swe
22	10418	6t9n	23	10534	6tni	24	10585	6ttu
25	10595	6tut	26	10617	6xt9	27	20145	6oo4
28	20146	6oo5	29	20189	6osy	30	20234	6p19
31	20249	6p4h	32	20254	6p5a	33	20259	6p62
34	20270	6p7v	35	20271	6p7w	36	20521	6pxm
37	20986	6v0b	38	21012	6v1i	39	21107	6v8o
40	21144	6vbu	41	21391	6vv5	42	3661	5no2
43	3662	5no3	44	3802	5of4	45	3885	6el1
46	3908	6eoj	47	4032	5lc5	48	4073	5lmn
49	4074	5lmo	50	4079	5lmt	51	4148	5m3m
52	4162	6ezo	53	4192	6f6w	54	4214	6fai
55	4241	6fe8	56	4272	6fki	57	4288	6fo2
58	4401	6i2x	59	4404	6i3m	60	4429	6i84
61	4588	6qm5	62	4589	6qm6	63	4593	6qma
64	4728	6r5k	65	4746	6r7x	66	4759	6r8f
67	4888	6ric	68	4889	6rid	69	4890	6rie
70	4917	6rla	71	4918	6rlb	72	4941	6rn3
73	4983	6rqj	74	7009	6ave	75	7041	6b3q
76	7065	6b7y	77	7090	6bf6	78	7334	6c23
79	7335	6c24	80	8958	6e1n	81	8960	6e1p
82	9258	6muw	83	9259	6mux	84	9695	6iok
85	9931	6k7g	86	9934	6k7i	87	9935	6k7j
88	9939	6k7l	89	9941	6k7m			

Supplementary Table 4. EMDB and PDB IDs for maps and models used for MC-EMmerNet validation

#	EMDB	PDB	#	EMDB	PDB	#	EMDB	PDB
1	0257	6hra	2	0499	6nsk	3	10401	6t8h
4	20849	6uqk	5	4611	6qp6	6	4646	6qvb
7	4733	6r69	8	4789	6rb9	9	7133	6bqv
10	7882	6dg7	11	8069	5i08	12	9112	6mgv
13	9298	6mzc	14	9374	6nhv			

Supplementary Table 5. EMDB and PDB IDs for maps and models used for testing and uncertainty calibration

#	EMDB	PDB	#	EMDB	PDB	#	EMDB	PDB
1	0282	6huo	2	0311	6hz5	3	10365	6t23
4	20220	6oxl	5	20226	6p07	6	3545	5mqf
7	4571	6qk7	8	4997	6rtc	9	7127	6bpq
10	8702	5vkq	11	9610	6adq			

Supplementary Table 6. EMDB and PDB IDs for maps and models in additional test dataset

#	EMDB	PDB	#	EMDB	PDB	#	EMDB	PDB
1	0193	6hcg	2	51108	9g71	3	44469	9bdt
4	35691	8isi	5	13234	7p6z	6	19999	9evd
7	17929	8pu6	8	0665	6oa9	9	10106	6s6u
10	33270	7xkt	11	3061	5a63	12	17225	8ovx
13	15037	7zz8	14	15205	8a64	15	14427	7z0l
16	17225	8ovx	17	15803	8b1r	18	16036	8bgu
19	28153	8ehw	20	26618	7una	21	15949	–
22	12654	7nyu	23	33888	7yk6	24	33394	7xqf

Supplementary Table 7. Details of MC-EMmerNet architecture

Layer	Layer Type	Output Shape	Kernel size	Stride	Parameter Count	Connected to
1	Input	$32^3 \times 1$	–	–	0	-
2	3D Conv+PReLU+GN	$32^3 \times 32$	5	1	4128	1
3	Dropout	$32^3 \times 32$	–	–	0	2
4	3D Conv+PReLU+GN	$32^3 \times 32$	5	1	128128	3
5	Dropout	$32^3 \times 32$	–	–	0	4
6	3D Conv+PReLU+GN	$16^3 \times 32$	5	2	128128	5
7	Dropout	$16^3 \times 32$	–	–	0	6
8	3D Conv+PReLU+GN	$16^3 \times 64$	5	1	256256	7
9	Dropout	$16^3 \times 64$	–	–	0	8
10	3D Conv+PReLU+GN	$16^3 \times 64$	5	1	512256	9
11	Dropout	$16^3 \times 64$	–	–	0	10
12	3D Conv+PReLU+GN	$8^3 \times 64$	5	2	512256	11
13	Dropout	$8^3 \times 64$	–	–	0	12
14	3D Conv+PReLU+GN	$8^3 \times 128$	5	1	1024512	13
15	Dropout	$8^3 \times 128$	–	–	0	14
16	3D Conv+PReLU+GN	$8^3 \times 128$	5	1	2048512	15
17	Dropout	$8^3 \times 128$	–	–	0	16
18	3D Conv+PReLU+GN	$4^3 \times 128$	5	2	2048512	17
19	Dropout	$4^3 \times 128$	–	–	0	18
20	3D Conv+PReLU+GN	$4^3 \times 128$	5	1	2048512	19
21	Dropout	$4^3 \times 128$	–	–	0	20
22	Trans-conv+Skip Concat	$8^3 \times 256$	5	2	2048128	21,14
23	3D Conv+PReLU+GN	$8^3 \times 128$	5	1	4096512	22
24	Dropout	$8^3 \times 128$	–	–	0	23
25	3D Conv+PReLU+GN	$8^3 \times 128$	5	1	2048512	24
26	Dropout	$8^3 \times 128$	–	–	0	25
27	3D Conv+PReLU+GN	$8^3 \times 128$	5	1	2048512	26
28	Dropout	$8^3 \times 128$	–	–	0	27
29	Trans-conv+Skip Concat	$16^3 \times 128$	5	2	1024064	28,8
30	3D Conv+PReLU+GN	$16^3 \times 64$	5	1	1024256	29
31	Dropout	$16^3 \times 64$	–	–	0	30
32	3D Conv+PReLU+GN	$16^3 \times 64$	5	1	512256	31
33	Dropout	$16^3 \times 64$	–	–	0	32
34	3D Conv+PReLU+GN	$16^3 \times 64$	5	1	512256	33
35	Dropout	$16^3 \times 64$	–	–	0	34
36	Trans-conv+Skip Concat	$32^3 \times 64$	5	2	256032	35,2
37	3D Conv+PReLU+GN	$32^3 \times 32$	5	1	256128	36
38	Dropout	$32^3 \times 32$	–	–	0	37
39	3D Conv+PReLU+GN	$32^3 \times 32$	5	1	128128	38
40	Dropout	$32^3 \times 32$	–	–	0	39
41	3D Conv+PReLU+GN	$32^3 \times 32$	5	1	128128	40
42	Dropout	$32^3 \times 32$	–	–	0	41
43	Trans-conv	$64^3 \times 16$	5	2	64016	42
44	3D Conv+PReLU+GN	$32^3 \times 8$	5	2	16032	43
45	Dropout	$32^3 \times 8$	–	–	0	44
46	Final 3D Convolution	$32^3 \times 1$	5	1	1001	45
-	Total parameters	-	-	-	22,875,161	–
