# Supplementary material for: Confidence-guided cryo-EM map optimisation with LocScale-2.0
Source: Nat Commun. 2026 Jul 17;17:8778. doi: 10.1038/s41467-026-75327-8 (PMC13493958; doi:10.1038/s41467-026-75327-8)
Supplement: Supplementary file 2 [file 41467_2026_75327_MOESM2_ESM.pdf]

nature portfolio

Corresponding author(s): Arjen J. Jakobi

Last updated by author(s): 19-05-2026

Reporting Summary

Nature Portfolio wishes to improve the reproducibility of the work that we publish. This form provides structure for consistency and transparency in reporting. For further information on Nature Portfolio policies, see our Editorial Policies and the Editorial Policy Checklist.

Statistics

For all statistical analyses, confirm that the following items are present in the figure legend, table legend, main text, or Methods section.

n/a	Confirmed	
☐	☒	The exact sample size (*n*) for each experimental group/condition, given as a discrete number and unit of measurement
☐	☒	A statement on whether measurements were taken from distinct samples or whether the same sample was measured repeatedly
☐	☒	The statistical test(s) used AND whether they are one- or two-sided<br>*Only common tests should be described solely by name; describe more complex techniques in the Methods section.*
☒	☐	A description of all covariates tested
☐	☒	A description of any assumptions or corrections, such as tests of normality and adjustment for multiple comparisons
☐	☒	A full description of the statistical parameters including central tendency (e.g. means) or other basic estimates (e.g. regression coefficient) AND variation (e.g. standard deviation) or associated estimates of uncertainty (e.g. confidence intervals)
☐	☒	For null hypothesis testing, the test statistic (e.g. *F*, *t*, *r*) with confidence intervals, effect sizes, degrees of freedom and *P* value noted<br>*Give P values as exact values whenever suitable.*
☒	☐	For Bayesian analysis, information on the choice of priors and Markov chain Monte Carlo settings
☒	☐	For hierarchical and complex designs, identification of the appropriate level for tests and full reporting of outcomes
☐	☒	Estimates of effect sizes (e.g. Cohen's *d*, Pearson's *r*), indicating how they were calculated

Our web collection on statistics for biologists contains articles on many of the points above.

Software and code

Policy information about availability of computer code

Data collection	Roodmus 0.0.35
Data analysis	UCSF ChimeraX v1.11, ModelAngelo v1.0.1, DeepEMhancer v0.16, EMReady v1.0, Phenix v2.0 , ToBvalid v0.9.7, REFMAC v 5.8, Servalcat v0.4.100

For manuscripts utilizing custom algorithms or software that are central to the research but not yet described in published literature, software must be made available to editors and reviewers. We strongly encourage code deposition in a community repository (e.g. GitHub). See the Nature Portfolio guidelines for submitting code & software for further information.

Data

Policy information about availability of data

All manuscripts must include a data availability statement. This statement should provide the following information, where applicable:

- Accession codes, unique identifiers, or web links for publicly available datasets
- A description of any restrictions on data availability
- For clinical datasets or third party data, please ensure that the statement adheres to our policy

All published data sets used for training, validation, testing and analysis were accessed and dowloaded from the EMDB and PDBe with accession codes specified in the Figure legends and Supplementary Tables. Data generated in this study are available from the 4TU.ResearchData repository (DOI: https://doi.org/10.4121/7fac93cc-d8f3-4f14-85a5-c7f1daa5c455)59. Pretrained MC-EMmerNet models (v0.3) can be accessed via Zenodo (DOI: https://zenodo.org/

records/8211668)60. Scripts used to generate figures shown in this study can be found at https://github.com/cryotud/publications. LocScale-2.0 is open-source software released under a BSD license and available at https://github.com/cryotud/locscale, where detailed documentation is also provided. Source data are provided with this manuscript.

Research involving human participants, their data, or biological material

Policy information about studies with human participants or human data. See also policy information about sex, gender (identity/presentation), and sexual orientation and race, ethnicity and racism.

Reporting on sex and gender	n/a
Reporting on race, ethnicity, or other socially relevant groupings	n/a
Population characteristics	n/a
Recruitment	n/a
Ethics oversight	n/a

Note that full information on the approval of the study protocol must also be provided in the manuscript.

Field-specific reporting

Please select the one below that is the best fit for your research. If you are not sure, read the appropriate sections before making your selection.

☒ Life sciences ☐ Behavioural & social sciences ☐ Ecological, evolutionary & environmental sciences

For a reference copy of the document with all sections, see nature.com/documents/nr-reporting-summary-flat.pdf

Life sciences study design

All studies must disclose on these points even when the disclosure is negative.

Sample size

No statistical method was used to predetermine sample size. Sample sizes were determined by the availability of suitable EMDB/PDB map–model pairs or EMDB half maps for which the respective LocScale-2.0 analyses could be performed, including generation of reliable FDR-based confidence masks, LocScale-2.0 reference maps and, where required, corresponding atomic or hybrid models.

For the physics-informed LocScale-2.0 validation, 83 EMDB entries were used to compare pseudomodel, atomic-model and hybrid-model representations, including analysis of window-averaged ADP correlations and model-to-map FSC curves. For automated model-building evaluation, 50 EMDB/PDB map–model pairs with reported FSC resolution better than 4.0 Å were used to compare ModelAngelo performance on deposited and LocScale-2.0-optimised maps.

For MC-EMmerNet/LocScale-FEM training, the dataset was derived from DeepEMhancer map–model pairs after excluding cases for which reliable confidence masks could not be generated. EMDB entries were grouped by 30% sequence-identity clusters, and training, validation and test sets were constructed to be mutually exclusive at this level; only one representative per sequence cluster was retained in the validation and test sets. Hybrid model maps were available for 114 entries, comprising 89 training maps, 14 validation maps and 11 independent maps reserved for testing and uncertainty calibration. Training examples consisted of 32 Å cubic subvolumes sampled from averaged unmodified EMDB half maps and paired LocScale-2.0 reference maps on a 24 Å grid, with a 4:1 ratio of signal-containing to noise-containing cubes.

Additional EMDB entries were selected for specific validation tasks. A set of 24 additional maps was used to assess contextual-density recovery, retention of non-protein components and pVDDT confidence-score behaviour. Quantitative comparison of contextual-density recovery against alternative map-optimisation methods was performed on 26 randomly selected EMDB maps using volume-recall analysis. Robustness of pVDDT calibration was further assessed in the rebuttal analyses using an independent calibration set of 50 randomly selected EMDB entries spanning a broad range of reported resolutions; pVDDT scores were compared on four additional EMDB entries not included in either calibration set, using 5,000 randomly sampled voxels per map within the calibration range.

Several other examples in the manuscript, including membrane-protein, subtomogram-averaging and ligand-containing maps, were selected as representative case studies to illustrate specific behaviours of the method, such as preservation of weak contextual density, interpretation of ligand density and visualisation of pVDDT confidence scores. These case studies were not used to define statistical sample sizes but to demonstrate method performance across biologically diverse reconstruction types. The dataset composition was further summarised in the rebuttal analysis, which reports that 15% of EMDB entries contained nucleic acids and 33% contained membrane or membrane-mimic density, with subset-specific fractions of 8–20% and 30–64%, respectively.

Data exclusions	No data were excluded in the analyses.
Replication	Replication is not applicable because this manuscript reports software that produces deterministic results. All results can be reproduced using the publicly available software and the specifications provided in the Methods section and Supporting Information.

Randomization	Randomization was not relevant because this was a retrospective computational study using pre-existing EMDB/PDB datasets. Samples were assigned to training, validation and test sets based on sequence-identity clustering to reduce redundancy and prevent information leakage, rather than by random allocation.
Blinding	Blinding was not necessary in this study because the analyses were performed on publicly available datasets using automated software workflows without human intervention.

Reporting for specific materials, systems and methods

We require information from authors about some types of materials, experimental systems and methods used in many studies. Here, indicate whether each material, system or method listed is relevant to your study. If you are not sure if a list item applies to your research, read the appropriate section before selecting a response.

Materials & experimental systems

n/a	Involved in the study
☒	☐ Antibodies
☒	☐ Eukaryotic cell lines
☒	☐ Palaeontology and archaeology
☒	☐ Animals and other organisms
☒	☐ Clinical data
☒	☐ Dual use research of concern
☒	☐ Plants

Methods

n/a	Involved in the study
☒	☐ ChIP-seq
☒	☐ Flow cytometry
☒	☐ MRI-based neuroimaging

Plants

Seed stocks	n/a
Novel plant genotypes	n/a
Authentication	n/a
