# Supplementary material for: Confidence-guided cryo-EM map optimisation with LocScale-2.0
Source: Nat Commun. 2026 Jul 17;17:8778. doi: 10.1038/s41467-026-75327-8 (PMC13493958; doi:10.1038/s41467-026-75327-8)
Supplement: Supplementary file 3 [file 41467_2026_75327_MOESM3_ESM.pdf]

nature portfolio

Peer Review File

Confidence-guided cryo-EM map optimisation with LocScale-2.0

Corresponding Author: Dr Arjen Jakobi

This file contains all reviewer reports in order by version, followed by all author rebuttals in order by version.

Version 0:

Reviewer comments:

Reviewer #1

(Remarks to the Author)
This manuscript presents LocScale-2.0, a computational framework aimed at improving the quality and interpretability of cryoEM 3-D reconstructions through locally adaptive sharpening and voxel-wise confidence estimation. The need for such approaches is well justified: cryoEM maps often exhibit spatially variable resolution, anisotropic signal falloff, and uneven noise distribution, leading to difficulties in applying global sharpening or interpreting weak local density. Methods capable of locally modulating map amplitudes and quantifying predictive uncertainty are increasingly essential as the field moves toward high-throughput, automated structure determination and model validation. The authors address these challenges by extending the original LocScale method to incorporate Bayesian inference and uncertainty quantification, moving beyond the limitations of the original framework to offer a potentially generalizable framework for confidence-aware map optimization. Overall, the quality of the manuscript is very high. It is clearly written, logically structured, and accessible to both technical and general audiences. While the work represents a valuable technical advance, several aspects of the methodology, validation, and user guidance might benefit from further clarification to ensure robustness, reproducibility, and broader adoption.

Sensitivity to Box Size and Object Volume Fraction
Although the authors emphasize that LocScale-2.0 operates in a locally adaptive manner, it remains unclear how the method behaves as a function of box dimensions relative to the macromolecular volume. For instance, in many reconstructions of small soluble proteins placed within large solvent boxes, or membrane proteins occupying only a fraction of the total volume, local priors and false-discovery rate (FDR) thresholds may be affected by the macromolecular fill-fraction. Although the authors evaluated numerous volumes with varying voxel sizes and box sizes, this concept was not explicitly evaluated.

It would be valuable for the authors to perform systematic tests varying the box and window sizes to evaluate the stability of local scaling and confidence estimation. For instance:
• Use large boxes (object < 30 % of volume) to test whether solvent-dominated priors bias sharpening toward over-filtering.
• Use tightly cropped boxes (object > 80 % of volume) to assess whether edge effects or over-regularization degrade performance.
• Include synthetic test volumes with increasing anisotropy (i.e., those observed from preferential orientation) or solvent padding to quantify how local variance estimates and map–model correlations change under these conditions.
These analyses would allow the authors to offer generalized recommendations for optimal box-to-object ratios and confirm whether the model is indeed insensitive to these boundary conditions.

Treatment and Interpretation of Refined B-factors
The manuscript introduces a predicted or refined local B-factor as part of the map optimization. While this approach may provide greater spatial granularity, Guinier-based analyses remain the conventional means for estimating global and local B-factors in cryoEM. The rationale for moving to a learned-B formalism should be expanded.
Specifically, do the predicted B-values reflect physically interpretable parameters, such as local disorder or atomic displacement, or are they better viewed as effective sharpening coefficients within the model? Quantitative comparisons between predicted and Guinier-derived B-factors would help clarify this relationship. If the main advantage is robustness rather than physical interpretability, that point should be explicitly stated.

Communication of Confidence (pVDDT-like) Scores
The introduction of voxel-wise predictive uncertainty represents a major step toward interpretable cryoEM map refinement. However, the practical utility of these "confidence maps" depends critically on how the values are scaled and communicated to users.
The authors should consider:
• Defining a standardized, normalized confidence metric (0–1 or 0–100 scale) analogous to pLDDT from AlphaFold, with fixed color bars for visual consistency.
• Providing calibration curves showing correlation between predicted confidence and independent quality metrics (local FSC, Q-score, or half-map reproducibility).
• Packaging the confidence maps as auxiliary MRC/CCP4 files with explicit metadata, ensuring that downstream software (ChimeraX, CCP-EM, Phenix) can display them without additional formatting.
A clear, standardized presentation of these scores will help users interpret confidence values intuitively and prevent misinterpretation of compositional or contrast-driven differences as uncertainty variations. Of note: I think this is a very important aspect of the map modification approach and enthusiastically support its inclusion in the outputs to the user, just need to make sure all users are fully aware of the implementation and how to evaluate.

Benchmarking, Parameter Guidance, and Computational Performance
Although the manuscript demonstrates improvements on several test cases, the examples appear biased toward high-quality, isotropic datasets. Providing clear parameter recommendations, such as default window sizes, recommended dropout rates, and stopping criteria, would greatly improve reproducibility and help less experienced users integrate the tool into existing pipelines.
Additionally, computational performance is not addressed. Runtime and memory requirements will strongly influence adoption. The authors should provide timing benchmarks for representative volumes (e.g., 128^3, 256^3, 384^3, 512^3 voxels) on common hardware, noting any linear or nonlinear scaling with box size. These benchmarks could be incorporated naturally into the box-size sensitivity analysis suggested above, allowing readers to evaluate both the computational scaling and the resulting map-quality scaling within the same framework.

Clarification of Bayesian Uncertainty Convergence
Because the confidence estimation relies on variational inference with Monte Carlo dropout, additional detail is needed on how convergence is assessed. Reporting the evolution of the evidence lower bound (ELBO), validation loss, and predictive-variance distribution over training epochs would clarify when the Bayesian approximation is stable. Furthermore, specifying how many stochastic forward passes are required for a reliable estimate of the mean and variance would provide practical guidance for computational efficiency.

Output Deliverables
It is currently unclear what specific data products are produced by LocScale-2.0. The manuscript should explicitly list the output files and their intended usage. For example:
• The sharpened or locally scaled map (MRC/CCP4)
• The voxel-wise confidence or uncertainty map
• Associated metadata (e.g., local B-factor volume, masks, run parameters, and log files)
• Are the dummy atom files available?
Including a schematic of the output hierarchy would clarify which maps should be deposited in EMDB versus used internally for model building.

Comparison to Other Density Modification Approaches
The authors compare LocScale-2.0 primarily to Phenix.autosharpen, yet omit direct comparison to Phenix.resolve_cryo_em (formerly resolve_em), which, unlike autosharpen, is also a density modification algorithm that incorporates local map–model priors and solvent flattening. Given that resolve_em and LocScale-2.0 share conceptual similarities in their treatment of local density weighting and prior-informed amplitude optimization, it would be important to understand why this comparison was not performed.
The authors should clarify whether such analyses were attempted, and if not, provide justification (e.g., differing input requirements, parameter incompatibilities, or computational limitations). Ideally, inclusion of one or two head-to-head comparisons—using identical half-maps and masks—would contextualize the claimed improvements relative to other modern density-modification methods and help delineate the specific advantages conferred by the Bayesian confidence framework introduced here.

Evaluation of Heterogeneity versus Local Resolution
One of the most promising potential uses of LocScale-2.0 lies in its ability to differentiate between regions of intrinsic structural heterogeneity and those of reduced local resolution arising from imaging or alignment limitations. The authors should explore this explicitly using test cases known to exhibit conformational variability, such as spliceosome assemblies, ribosomal intermediates, or viral capsid expansion states where compositional and conformational heterogeneity are well characterized in the literature.
Applying LocScale-2.0 to such systems could demonstrate whether voxel-wise confidence values correlate more strongly with true conformational variability (e.g., multiple states or flexible domains) rather than simply local resolution estimates. This would not only validate the interpretability of the uncertainty metric but also help establish the method's value as a diagnostic tool for distinguishing dynamic from static disorder within complex macromolecular machines.

Minor Comments
• Clarify whether reported confidence values are absolute or relative within each reconstruction; this distinction is critical for

inter-map comparison.
• Ensure consistent terminology throughout: if “confidence-guided map” and “uncertainty-weighted map” refer to the same output, state this explicitly.
• Including example ChimeraX session files or command-line scripts would enhance reproducibility.

Summary
LocScale-2.0 represents an important and technically ambitious evolution of local scaling methods, incorporating Bayesian confidence estimation into cryoEM map refinement.

(Remarks on code availability)
My system administrator was able to access, install, and run the code on our system.

Reviewer #2

(Remarks to the Author)
The manuscript by Bharadwaj et al. represents a significant improvement over their successful pioneer work in map enhancement, LocScale (1.0). The original work had a key limitation in that it required substantial prior knowledge of the structure to be enhanced (or a closely related one), a shortcoming that has been addressed in this new "version." Additionally, the authors introduce a deep learning "version" of their method, which offers substantial speed, enabling them to provide some form of confidence limits.

The manuscript is generally well written, although not always easy to read, despite the large amount of information presented. I suggest some ways to improve it.

The main body of the manuscript could be more schematic while conveying some key information more clearly. In particular, in the main text it is indicated that instead of the prior information demanded by their older method, now they use “First-principle expectations and data-driven approaches”… which, as such, is not very informative for the average user (in the Methods section, the description is clear, with REFMAC as a central approach). Could the authors present a bit more in the central part of the work, what these “first principles” are?

On the Figures, in general, they are so loaded with data and the labels are so small, that in the printed copy of the manuscript many labels are unreadable.

Then, in the deep learning section, the motivation is clear, and the authors explain their calibration approach well. However, since the confidence measure comes from repeatedly applying the same method, I wonder if they might have a systematic error or bias that calibration cannot fix.

In the method section, I do not understand the relationship with DeepEMhancer (why does the training set correspond to the one used by that method?). Much has changed since DeepEMhancer was introduced/trained

Finally, although I understand that the method works specifically in Fourier space and not in real space, I suggest that popular quality measures like Q-factor could also be included, perhaps as Supplementary Material.

(Remarks on code availability)

Reviewer #3

(Remarks to the Author)
The authors present an AI method to modify the density of a cryoEM reconstruction. The method relies on a previous physics-based method to adjust the amplitudes of cryoEM maps to better represent the included mass of the particle. It makes use of filling the estimated volume with pseudo-atoms, thus not relying on a specific model. However, as the authors state, the pseudo-atomic approach is useful at lower spatial frequencies and does not capture the high resolution details. To extend resolution, the method still relies on specific models, and may still retain some bias. Additionally, the method relies strongly on adequate calibration and numerous adjustments, which depends on the parameters the user chooses. This drastically increases the likelihood of generating artifacts, as well as complicating interpretation. The main problem is that it depends on a series of interpretations, where introducing some bias in one step propagates and magnifies in the next. Therefore, in careful hands, this method could be used to generate details in maps that could be subsequently tested by other methods. The possibility of generating artifacts should be addressed by the authors where they discuss the limitations. With these issues raised, I believe it is a useful study that warrants publication.

I find the results very complicated to interpret and was not able to properly comment on the details. I therefore focused on the methods:

Line 669:
The density is in g/cm3 and Avogadro's constant is in #/mol, but the volume is presumably in Å3 and the average atomic mass is in Da. It would be better to have relatable units, such as Da/Å3 for density.

Line 744:
I don't understand the relevance of REFMAC here. Please clarify.

Line 814:
Where did these "LocScale-2.0 reference maps" come from? Were they calculated from existing models?

Line 847:
What does dropout mean? How does it compare with the Metropolis criterion?

(Remarks on code availability)
Because of time limitations and the complicated nature of the methods, I have not examined the code.

Reviewer #4

(Remarks to the Author)
The manuscript presents LocScale-2.0, a map-optimization framework aimed at handling spatially heterogeneous cryo-EM reconstructions by enhancing local detail without requiring prior structural knowledge. The approach combines (i) a physics-informed, model-independent procedure that generates local references and performs local Fourier-amplitude scaling, and (ii) a deep-learning approach (MC-EMmerNet) trained to emulate the physics-informed output and to produce feature-enhanced maps (LocScale-FEM) alongside voxel-wise confidence scores (pVDDT).

Overall, I find the contribution potentially high-impact and practically useful, especially for reconstructions with contextual density (e.g. membranes, glycans) where common sharpening/denoising may suppress biologically relevant features. It appears to improve on DeepEMhancer, especially by providing a confidence-guided deep-learning post-processing framework. The manuscript is also clearly written. However, in my view the manuscript cannot be accepted before major revision, because the current training/evaluation setup does not yet define a sufficiently realistic scope of validity for the deep-learning component nor demonstrate robust generalizability of the confidence estimates.

Major concerns:

1) Scope and realism of the data augmentation strategy

The augmentation strategy (random rotations; random sharpening/blurring using B-factors 0–400 $Å^2$; Gaussian low-pass filtering) mainly introduces isotropic, smoothly varying spectral changes. In contrast, loss of high-resolution information in experimental cryo-EM maps is often anisotropic, spatially heterogeneous, and non-Gaussian, driven by residual CTF effects, preferred orientation, beam-induced motion, masking/reconstruction artifacts, and locally weak/partially ordered components.

While the chosen augmentations are described clearly, the manuscript does not evaluate whether they capture these experimentally relevant distortions. As a result, the network may be optimized for patterns consistent with the LocScale-2.0 reference maps, while its behavior on maps dominated by anisotropy and locally heterogeneous signal remains insufficiently characterized. In such cases, the physics-informed, phase-conservative workflow may be the safer option. Consequently, a more systematic evaluation of the method's correctness on maps exhibiting pronounced anisotropy and locally heterogeneous signal would be necessary to delineate its range of validity and reliability.

2) Size, diversity, and characterization of the training and evaluation datasets (incl. ligands)

The authors are commended for listing all EMDB/PDB entries used. However, the deep model is trained on 114 EMDB/PDB entries, which is a limited number of independent maps. Although overlapping 32 Å sub-volumes substantially increase the number of training samples, many patches originate from the same maps, reducing the effective diversity of the dataset. The manuscript also does not report structural redundancy (e.g., homologs, repeated architectures, similar complexes in different states), which could inflate apparent generalization.

The uncertainty calibration test set comprises 11 maps, which may limit robustness across heterogeneous targets. In addition, the manuscript would benefit from a statistical summary of dataset properties (resolution distribution, modality/instrumentation, biological classes), and stratified performance across resolution ranges; currently quantitative analysis focuses largely on ~3–6 Å leaving performance at lower resolutions insufficiently characterized.

A clearer assessment of dataset diversity and redundancy, together with stratified evaluations across resolutions and structural classes, would help delineate the range of applicability and generalizability of the method.

3) Ligands: I appreciate that ligands are considered, but I'm concerned about whether there is sufficient training diversity. A small number of maps can provide extensive protein-density examples, but ligands are a small fraction of voxels/models, so generalisation may be weaker precisely where over-interpretation risk is highest.

Minor point

I found one typo: “interpretatioo” → “interpretation”.

(Remarks on code availability)

Version 1:

Reviewer comments:

Reviewer #1

(Remarks to the Author)
I thank the authors for their thorough, thoughtful, and very clearly articulated responses to all points raised. The additional analyses and clarifications substantially strengthen the manuscript, and I am fully supportive of the revised version. Looking forward to using the software in future work.

(Remarks on code availability)

Reviewer #2

(Remarks to the Author)
The authors have addressed all the comments we raised appropriately. They have also introduced further modifications to the manuscript, which have increased its clarity and accessibility, producing a very good and useful work for the cryoEM field. As very minor points, the authors might consider introducing the word "into RELATIVELY structural..." (Page 1, line 39)

J.M. Carazo and J.L. Vilas

(Remarks on code availability)
Although we have checked the existence of the repository itself

Reviewer #3

(Remarks to the Author)
The authors gave detailed responses arguing that they minimized the number of decisions users have to make. However, that means they made the decisions in code with the same possibilities of bias. I am therefore not convinced that their ML code would be immune to the typical hallucinations of AI. I would consider only the physics-based method to give reliable results. Any further extension suffers the same problems as density modification techniques taken too far. I'm therefore no comfortable with accepting the manuscript.

(Remarks on code availability)

Reviewer #4

(Remarks to the Author)
I am happy with the revision. I think the paper is ready to be published in Nat Comm.

(Remarks on code availability)

Reviewer #5

(Remarks to the Author)
I co-reviewed this manuscript with one of the reviewers who provided the listed reports. This is part of the Nature Communications initiative to facilitate training in peer review and to provide appropriate recognition for Early Career Researchers who co-review manuscripts.

(Remarks on code availability)
The authors have address all questions and comments we had in our previous review. I consider the manuscript reday for its publication

Open Access This Peer Review File is licensed under a Creative Commons Attribution 4.0 International License, which permits use, sharing, adaptation, distribution and reproduction in any medium or format, as long as you give appropriate credit to the original author(s) and the source, provide a link to the Creative Commons license, and indicate if changes were made.
In cases where reviewers are anonymous, credit should be given to 'Anonymous Referee' and the source.
The images or other third party material in this Peer Review File are included in the article's Creative Commons license, unless indicated otherwise in a credit line to the material. If material is not included in the article's Creative Commons license and your intended use is not permitted by statutory regulation or exceeds the permitted use, you will need to obtain permission directly from the copyright holder.
To view a copy of this license, visit https://creativecommons.org/licenses/by/4.0/

Rebuttal NCOMMS-25-79735

We would like to thank all four reviewers for the positive assessment of our work and their feedback. We provide a point-by-point response to the individual comments below.

Reviewer #1

This manuscript presents LocScale-2.0, a computational framework aimed at improving the quality and interpretability of cryoEM 3-D reconstructions through locally adaptive sharpening and voxel-wise confidence estimation. The need for such approaches is well justified: cryoEM maps often exhibit spatially variable resolution, anisotropic signal falloff, and uneven noise distribution, leading to difficulties in applying global sharpening or interpreting weak local density. Methods capable of locally modulating map amplitudes and quantifying predictive uncertainty are increasingly essential as the field moves toward high-throughput, automated structure determination and model validation. The authors address these challenges by extending the original LocScale method to incorporate Bayesian inference and uncertainty quantification, moving beyond the limitations of the original framework to offer a potentially generalizable framework for confidence-aware map optimization. Overall, the quality of the manuscript is very high. It is clearly written, logically structured, and accessible to both technical and general audiences. While the work represents a valuable technical advance, several aspects of the methodology, validation, and user guidance might benefit from further clarification to ensure robustness, reproducibility, and broader adoption.

Thank you for your appreciation of our work and the useful comments.

Although the authors emphasize that LocScale-2.0 operates in a locally adaptive manner, it remains unclear how the method behaves as a function of box dimensions relative to the macromolecular volume. For instance, in many reconstructions of small soluble proteins placed within large solvent boxes, or membrane proteins occupying only a fraction of the total volume, local priors and false-discovery rate (FDR) thresholds may be affected by the macromolecular fill-fraction. Although the authors evaluated numerous volumes with varying voxel sizes and box sizes, this concept was not explicitly evaluated.

It would be valuable for the authors to perform systematic tests varying the box and window sizes to evaluate the stability of local scaling and confidence estimation. For instance:

- *Use large boxes (object < 30 % of volume) to test whether solvent-dominated priors bias sharpening toward over-filtering.*
- *Use tightly cropped boxes (object > 80 % of volume) to assess whether edge effects or over-regularization degrade performance.*
- *Include synthetic test volumes with increasing anisotropy (i.e., those observed from preferential orientation) or solvent padding to quantify how local variance estimates and map–model correlations change under these conditions.*

These analyses would allow the authors to offer generalized recommendations for optimal box-to-object ratios and confirm whether the model is indeed insensitive to these boundary conditions.

We thank the reviewer for raising this point. The question refers to the behaviour of two conceptually distinct components of the LocScale-2.0 workflow that must be considered separately:

(1) the generation of FDR-controlled confidence masks, and
(2) the use of local priors and windowed Fourier-amplitude scaling during LocScale optimisation.

FDR confidence estimation: The behaviour of LocScale-2.0 regarding varying box size and object-to-box ratio follows directly from the statistical framework of FDR-controlled confidence estimation introduced in the original work on FDR thresholding (Beckers et al., PMID 30713700). In this framework, the null (noise) distribution is estimated from explicitly defined background regions, which in principle decouples confidence estimation from object occupancy within the reconstruction box. The robustness of FDR thresholding therefore primarily depends on the availability of sufficiently large, signal-free background regions. By default, LocScale-2.0 places noise-sampling boxes for FDR thresholding at the periphery of the reconstruction volume in all spatial directions. As explained in the Methods, the edge length of each noise sampling box is set to 10% of the corresponding reconstruction dimension, such that number of voxels used for noise estimation scales proportionally with the overall reconstruction volume. For filaments, noise sampling in the z direction must be omitted and this is implemented in the procedures. We note that the above assumptions can be more difficult to satisfy in subtomogram averages from in-cell cryo-ET reconstructions, where molecular signal may extend into regions that would otherwise serve for background sampling. In such cases, LocScale-2.0 allows manual coordinate adjustment for the placement of noise sampling boxes to ensure valid noise estimation.

Local scaling and window size (LocScale-2.0 map optimisation): Window size (where "window" refers to a rolling window) is relevant only for the LocScale optimisation step, where local Fourier-amplitude scaling is performed using reference maps. The windowed Fourier-amplitude scaling step operates exclusively within the FDR-derived confidence mask. Consequently, solvent-dominated regions outside the mask do not influence local priors or amplitude scaling. The window size affects the spatial locality of spectral estimation but is not dependent on the object-to-box ratio. Its behaviour and effect on the sharpening outcome have been systematically investigated in previous work (Jakobi et al., PMID 29058676; Bharadwaj & Jakobi, PMID 35938593), where we showed that performance is stable across a broad range of window sizes and that automated parameter selection is sufficient in practice. While window size can be freely adjusted by the user, LocScale-2.0 includes automated procedures to select default values without user input which are based on empirical estimates of our previous work.

Anisotropy: The reviewer also raises the case of anisotropic reconstructions arising from preferential particle orientation, where signal strength can vary across spatial directions. In this scenario, anisotropy affects the signal distribution in Fourier space, but the background noise arising from shot noise and structural noise remains approximately isotropic. Since FDR thresholding estimates the null distribution from explicitly defined solvent regions rather than from global map statistics, anisotropy in the molecular signal does not inherently bias confidence estimation, provided that the background sampling regions remain free of signal contamination as discussed above.

To illustrate this behaviour, we generated synthetic micrographs from SARS-CoV2 spike protein using our software Roodmus (Joosten et al., PMID 39404610) from which we obtained reconstructions either from particles in random orientations or confined in a very narrow tilt range (θ>80; **Figure R1**). In both cases, the FDR-derived confidence mask remains well confined to molecular density. Importantly, in the strongly anisotropic example shown, the FDR-derived confidence mask is itself elongated along the direction of poor orientation sampling. This demonstrates that the mask adapts to the anisotropic signal envelope. In other words, even weak but statistically supported density along the poorly sampled direction is retained. In the LocScale2.0-optimised map from the limited tilt range reconstruction, this anisotropy is attenuated. This, again, is an expected feature of the local scaling procedure, which involves spherical averaging in Fourier space and thus mitigates the directional anisotropy of the signal in the optimised map.

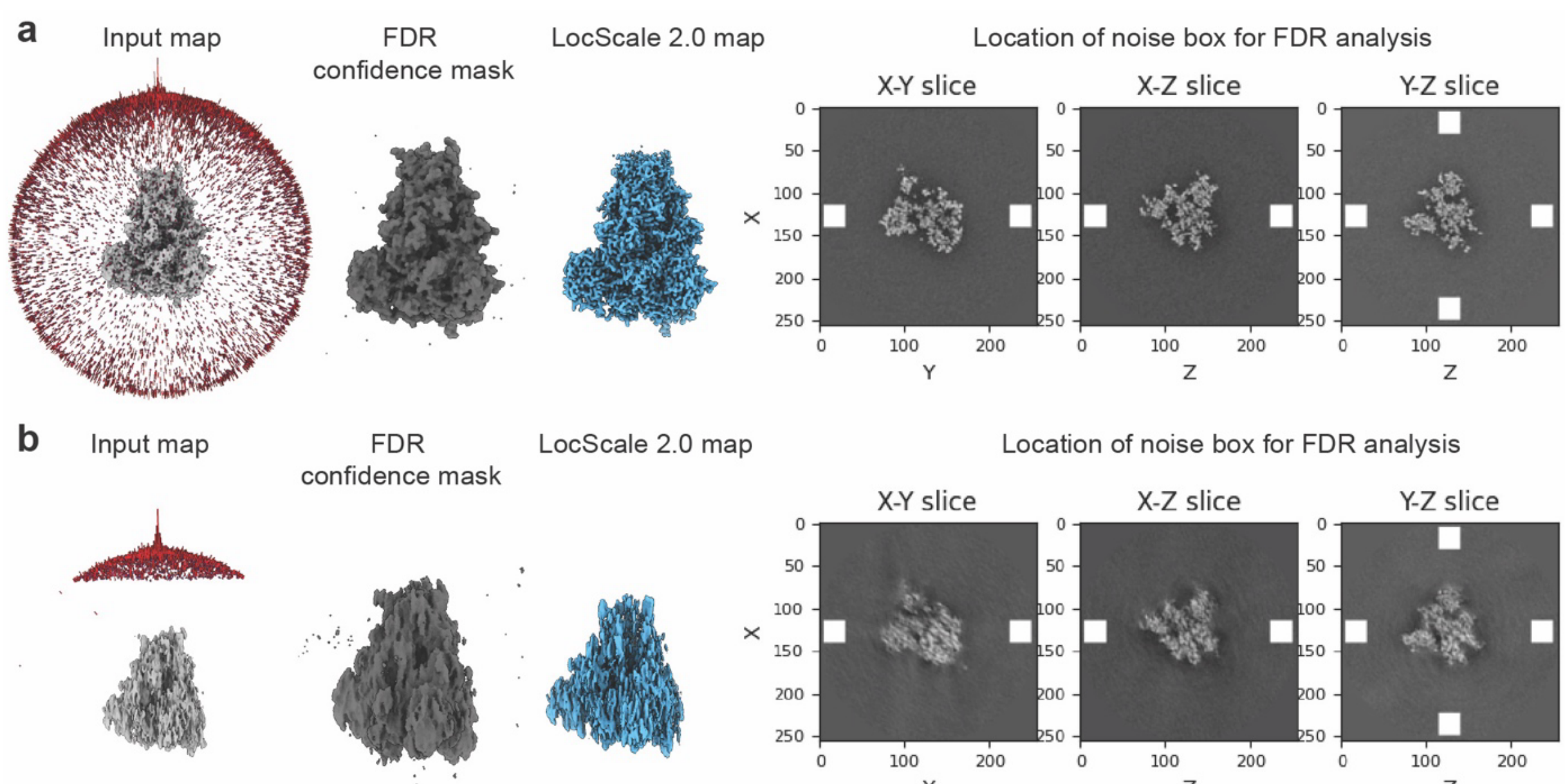

Figure R1 | Effect of anisotropy on FDR confidence masking and LocScale-2.0 optimisation. (a) Isotropic orientation sampling. Input map, corresponding FDR-derived confidence mask, and LocScale-2.0 optimised map are shown. Orthogonal slices indicate the automatically placed noise-sampling boxes (white squares) used for estimation of the null distribution during FDR thresholding. (b) Same analysis performed on a reconstruction exhibiting strong directional anisotropy (θ>80). The FDR-derived confidence mask is visibly elongated along the direction of missing orientations, demonstrating that statistical masking adapts to the anisotropic signal.

The manuscript introduces a predicted or refined local B-factor as part of the map optimization. While this approach may provide greater spatial granularity, Guinier-based analyses remain the conventional means for estimating global and local B-factors in cryoEM. The rationale for moving to a learned-B formalism should be expanded. Specifically, do the predicted B-values reflect physically interpretable parameters, such as local disorder or atomic displacement, or are they better viewed as effective sharpening coefficients within the model? Quantitative comparisons between predicted and Guinier-derived B-factors would help clarify this relationship. If the main advantage is robustness rather than physical interpretability, that point should be explicitly stated.

The rationale for using refined atomic displacement factors as estimates for local B-factors is described in detail in our prior work (Jakobi et al., PMID 29058676; Bharadwaj & Jakobi, PMID 35938593). In brief, local Guinier (more precisely, Wilson-type) B-factor estimation relies on linear regression of log-amplitudes from spherically averaged Fourier coefficients and becomes increasingly noisy and unstable as the spatial support of the local region decreases. In addition, such approaches provide no mechanism to enforce smooth and physically meaningful variation of B-factors along polypeptide or nucleic acid chains. In principle, B-factors can be expected to vary smoothly along connected atoms and residues, reflecting correlated atomic motion and structural flexibility. Map-based local Guinier analysis, however, operates in spatial windows in volume space, without a structural support, and therefore is insensitive to molecular connectivity. Spatially adjacent but structurally unconnected regions such as rigid and flexible domains in proximity may be averaged together, leading to underestimation of B-factors in flexible regions and overestimation in rigid ones. By contrast, refining local atomic displacement parameters against the experimental map provides a more robust estimate of local signal decay that respects molecular connectivity and therefore correlated motion, because the physical restraint framework enforces correlations between bonded atoms rather than between spatially adjacent but unconnected regions. In the context of LocScale-2.0, these refined B-factors are therefore best interpreted as effective local attenuation parameters of the spectral signal amplitudes. Their primary role is to provide robust and spatially adaptive scaling factors for local optimisation of Fourier amplitudes. In the LocScale-FEM framework, these local B-factors are learned directly from physics-informed LocScale-2.0 target maps. As shown in **Figure R2a-b**, we observe strong agreement between learned local B-factors and those obtained from hybrid atomic-model refinement (Pearson R≈0.9 for the representative case; consistently high correlations across the test set). This is confirmed by the spectral preservation of the local Fourier amplitudes between predicted and target maps shown in Supplementary Figure S6l. Together, this demonstrates that the network reproduces the effective signal decay signatures encoded in the refined B-factors of physics-informed procedure. Thus, these learned B-factors retain the same interpretation: they are robust local attenuation parameters used for adaptive Fourier amplitude scaling that correlate with atomic/structural disorder but should not be interpreted as direct physical measurements. We re-emphasise that the primary motivation for moving away from Guinier-based local B-factor estimation toward refined B-factors and, by extension this learned-B formalism, is robustness to overfitting and the improved locality of the optimisation.

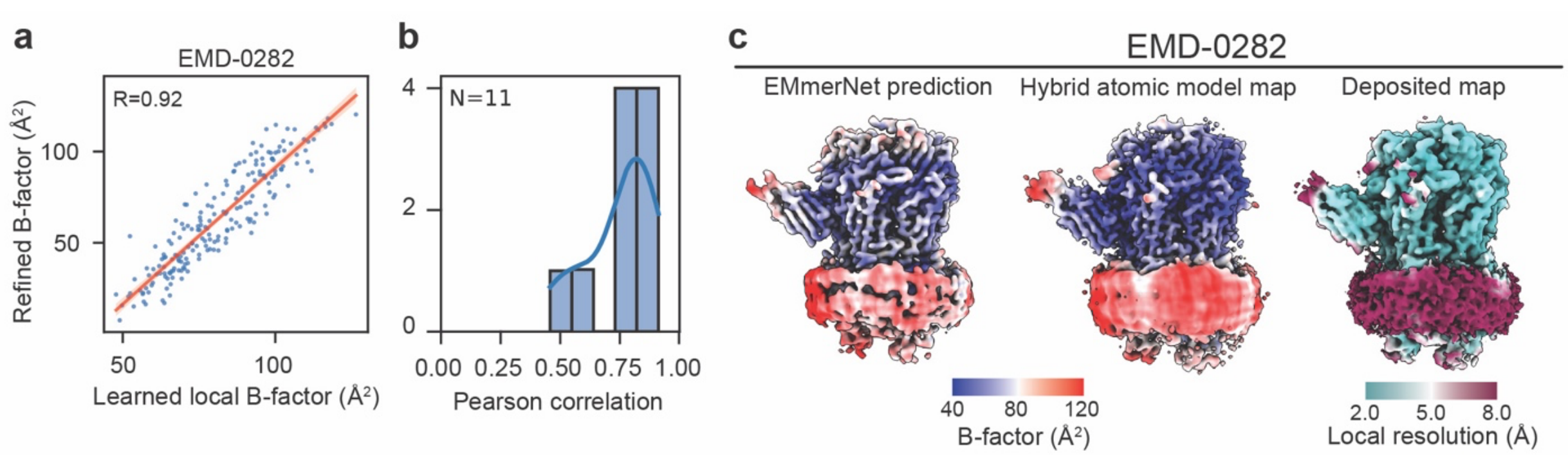

Figure R2 | Correlation of refined and predicted B-factors. (a) Correlation between learned local B-factors predicted by EMmerNet and refined B-factors obtained from hybrid atomic-model refinement in LocScale-2.0 shown for EMD-0282. Each data point represents the average B-factor of a local region; Pearson correlation coefficient is indicated. (b) Distribution of Pearson correlation coefficients across 11 EMDB-derived test maps demonstrates consistent agreement between learned and refined local B-factors. (c) Representative example (EMD-0282) comparing EMmerNet-predicted local B-factors, refined B-factors mapped onto the cryo-EM density map. Predicted and refined B-factor distributions show similar spatial patterns, indicating that EMmerNet reproduces the effective signal-decay of the physics-informed B-factor refinement. The corresponding unfiltered density coloured by local resolution is also shown and illustrates that both refined and predicted

Communication of Confidence (pVDDT-like) Scores

The introduction of voxel-wise predictive uncertainty represents a major step toward interpretable cryoEM map refinement. However, the practical utility of these "confidence maps" depends critically on how the values are scaled and communicated to users. The authors should consider:

• Defining a standardized, normalized confidence metric (0–1 or 0–100 scale) analogous to pLDDT from AlphaFold, with fixed color bars for visual consistency.

As detailed in the main text (Figures 5-7, Figures S7-S8) and Methods, the pVDDT score is already a calibrated and standardised confidence metric derived from a rescaled z-score, associated with a pLDDT-like colour map. Rather than mapping the values onto a 0-1 or 0-100 scale, we deliberately retain both positive and negative values for this confidence metric because the metric is designed to capture the direction as well as the magnitude of statistically significant deviations between the feature-enhanced prediction and the locally amplitude-matched baseline map. Positive and negative pVDDT values therefore have distinct interpretive meaning: positive scores indicate predicted densities stronger than expected from the baseline, whereas negative scores highlight regions where the prediction is weaker than supported by the experimental phases. Collapsing this signed information onto a strictly positive scale would remove this directional interpretability and reduce the utility of the metric. For visual consistency across datasets, we instead define reproducible interpretive ranges (e.g., –80 to +80 as reliable; *abs*(pVDDT) > 80 as statistically significant deviations), which are stable across maps due to the underlying calibration procedure (see also response to Reviewer 4 on calibration). We use a divergent color map to illustrate the confidence maps [see manuscript Figures 5-7, Figures S7-S8] and ChimeraX scripts to facilitate their visualisation by the users are a standard output of the program.

• Providing calibration curves showing correlation between predicted confidence and independent quality metrics (local FSC, Q-score, or half-map reproducibility).

We thank the Reviewer for this question, as it provides an opportunity to clarify what the pVDDT confidence score represents and what it does not measure. The pVDDT score is not a measure of intrinsic map quality of the optimised cryo-EM map, nor does it aim or claim to quantify local resolution or map interpretability. Instead, pVDDT measures the confidence in the prediction itself; specifically, the statistical significance of voxel-wise deviations between the feature-enhanced map and a phase-conservative, amplitude-matched baseline. In other words, pVDDT quantifies predictive reliability (i.e., the confidence in whether newly introduced features are statistically supported relative to the baseline), rather than absolute map correctness or resolution.

For this reason, direct calibration of pVDDT against conventional map-quality metrics such as local FSC or Q-scores would conflate conceptually different quantities. We are aware that Q-scores have been used in other publications reporting map optimisation methods to support claims of map improvement. However, we consider their use inappropriate in this context, as they represent a form of circular validation. Q-scores quantify how closely the local density around an atom matches an idealised, Gaussian-like atomic density. For map optimisation methods trained on model-derived target maps such as DeepEMhancer or EMReady improved Q-scores are therefore an expected consequence of the training objective rather than independent evidence of correctness or map improvement. This holds, indirectly, also for LocScale-FEM.

We do agree, however, that independent validation of map improvement would be useful. To address this, we performed additional half-map cross-validation experiments (**Figure R3**). Specifically, we generated LocScale-FEM maps independently from each half-map and computed cross-FSC between each feature-enhanced map and the opposite, independently reconstructed and unprocessed half-map. The same analysis was performed for amplitude-scaled baseline maps. Because each feature-enhanced map is derived from only one half-dataset, this procedure directly tests whether enhanced features are reproducible across independent data. Therefore, any increase in correlation of each feature-enhanced map with the respective independent half map must arise from signal features that are supported by both independent datasets. This logic provides a stringent test: if LocScale-FEM were hallucinating features specific to one half-map, the averaged cross-FSC against the independent half-map would decrease. If LocScale-FEM selectively enhances genuine, but weak, signal that is present in both half maps, the averaged cross-FSC will be preserved or increased. Because feature-enhancement primarily affects high resolution, we evaluate the averaged FSC beyond spatial frequencies of 0.2 Å^{-1} (>5 Å resolution). Across the tested EMDB entries, we observe that feature-enhanced maps maintain and frequently improve cross-half-map FSC relative to regular half maps FSCs and cross-FSCs of amplitude-scaled

baseline maps. This demonstrates that the enhanced features are generally supported by independent experimental signal. This analysis is now included in the revised manuscript (Supplementary Figure S7).

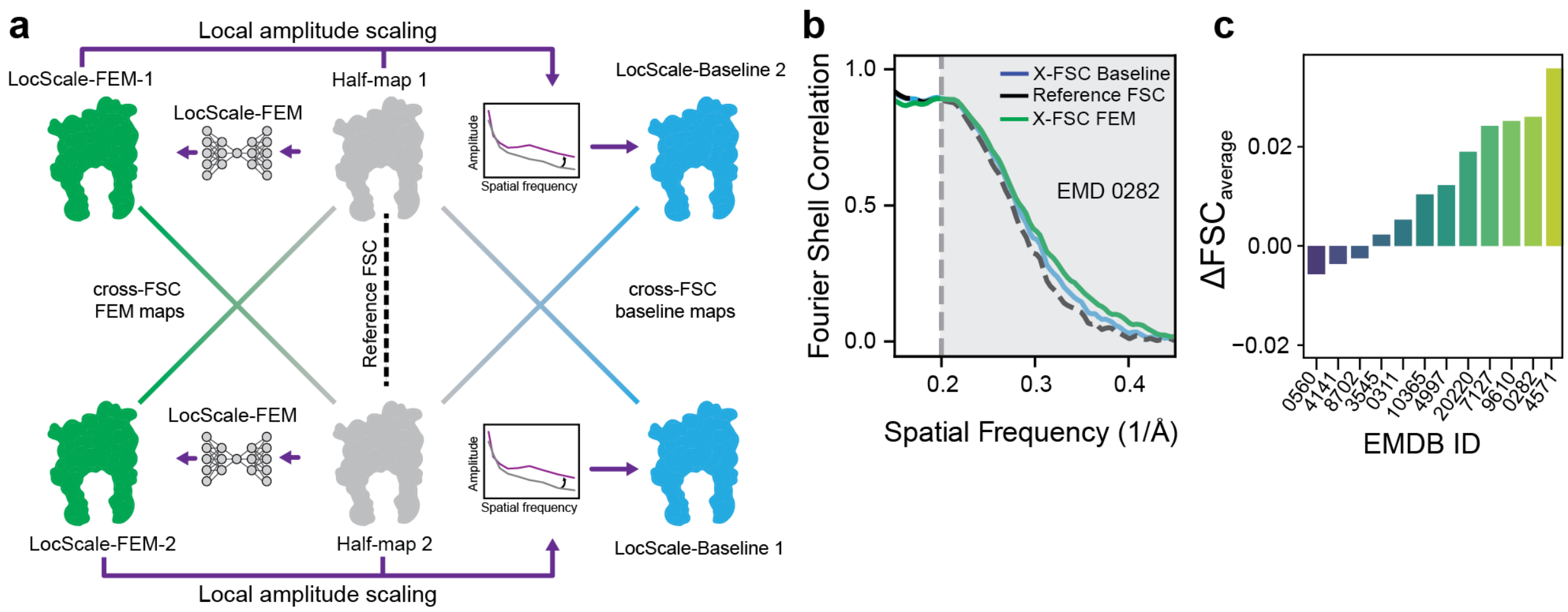

Figure R3 | Validation of LocScale-FEM map improvement. (a) Schematic of the cross-validation strategy. Each experimental half-map is processed independently to generate two feature-enhanced maps (LocScale-FEM-1 and LocScale-FEM-2), while corresponding amplitude-scaled baseline maps are generated in parallel. Cross-FSC is then computed between each processed map and the independent opposite half-map (e.g., LocScale-FEM-1 vs. Half-map 2). Because each feature-enhanced map is derived from only one half-dataset, this procedure directly tests whether enhanced features are supported by independent data. (b) Representative cross-FSC curves for EMD-0282 comparing baseline (blue) and LocScale-FEM (green) maps against the independent half-map reference (black). LocScale-FEM preserves or improves cross-half map agreement across spatial frequencies. (c) Average difference in cross-FSC ($\Delta FSC_{average}$) across a representative set of EMDB entries. Predominantly positive values indicate that feature-enhanced maps frequently improve agreement with independent data.

• *Packaging the confidence maps as auxiliary MRC/CCP4 files with explicit metadata, ensuring that downstream software (ChimeraX, CCP-EM, Phenix) can display them without additional formatting.*

A clear, standardized presentation of these scores will help users interpret confidence values intuitively and prevent misinterpretation of compositional or contrast-driven differences as uncertainty variations. Of note: I think this is a very important aspect of the map modification approach and enthusiastically support its inclusion in the outputs to the user, just need to make sure all users are fully aware of the implementation and how to evaluate.

We thank the reviewer for highlighting the importance of clearly presenting confidence scores, and we agree that transparent and standardised visualisation of confidence information is essential for correct interpretation of map modifications. As described in the manuscript and detailed in the online documentation for LocScale 2.0 [https://cryotud.github.io/locscale/], pVDDT confidence maps are provided as a standard output in MRC format and LocScale outputs ChimeraX session file (.cxs) that loads input, optimised and baseline maps and automatically maps the pVDDT scores onto the optimised maps. The manuscript's Methods section and accompanying documentation also explain how confidence scores can be directly mapped onto LocScale-FEM maps using a simple ChimeraX command. Moreover, visualisation of confidence-coloured LocScale-FEM maps is implemented as a standard final step of each LocScale-FEM run within CCPEM-Doppio workflows, ensuring that users are routinely exposed to these confidence representations. In addition, pVDDT confidence scores can be mapped in Moorhen using the *Map Tools → Colour By Other Map* function. We have added this information to the manuscript.

Benchmarking, Parameter Guidance, and Computational Performance

Although the manuscript demonstrates improvements on several test cases, the examples appear biased toward high-quality, isotropic datasets. Providing clear parameter recommendations, such as default window sizes, recommended dropout rates, and stopping criteria, would greatly improve reproducibility and help less experienced users integrate the tool into existing pipelines.

We thank the reviewer for this suggestion. Detailed documentation for the use of LocScale-2.0, including recommended parameter settings and explanations of all user-definable parameters, is provided in the online documentation (https://cryotud.github.io/locscale/), which is referenced in the manuscript. This documentation includes practical

guidance on all parameters relevant for routine use of the software. Importantly, LocScale-2.0 is designed to operate fully automatically in typical use cases. While all parameters can in principle be adjusted by the user, they all have defaults either estimated on the fly or based on empirically derived settings. As a result, routine map optimisation generally does not require manual parameter tuning.

The present manuscript is primarily intended to describe the physical basis and algorithmic implementation of LocScale-2.0 and as an account of its performance. For this reason, we opted not to include extensive usage instructions or the parameter guidelines within the main text, as these would substantially expand the methodological sections and shift the focus toward a user protocol rather than the underlying methodological advances. To address the reviewer's concern regarding accessibility for new users, we have clarified in the revised manuscript that practical recommendations and parameter explanations are available in the online documentation, where they can be maintained and updated alongside future software releases.

Additionally, computational performance is not addressed. Runtime and memory requirements will strongly influence adoption. The authors should provide timing benchmarks for representative volumes (e.g., 128^3, 256^3, 384^3, 512^3 voxels) on common hardware, noting any linear or nonlinear scaling with box size. These benchmarks could be incorporated naturally into the box-size sensitivity analysis suggested above, allowing readers to evaluate both the computational scaling and the resulting map-quality scaling within the same framework.

A breakdown of runtime for different map sizes was given in Table S1 of the original manuscript. We expanded this section and now include detailed runtimes for the most demanding LocScale-2.0 modality (LocScale-FEM map optimisation) for increasingly large molecular volumes. Map optimisation on typical maps will generally complete in a few minutes on standard hardware. Even for very large maps, LocScale-FEM optimisation typically completes in less than 1 hour. We have included these data in the revised manuscript (Tables S2).

EMDB / N	Box size (voxels)	Unit cell (Å)	Volume ($\times 10^6$ Å^3)	Compute time (s)	Notes
4997	232	249.4	15.5	$\sim$280	example
7127	256	335.3	37.7	$\sim$530	example
8702	400	486.2	114.9	$\sim$1200	example
3545	360	572.4	187.5	$\sim$4000	example
N=4	–	200–250	8–15	350 ± 200	mean $\pm$ s.d.
N=7	–	250–400	15–64	600 ± 300	mean $\pm$ s.d.
N=3	–	400–600	64–216	2600 ± 1150	mean $\pm$ s.d.

Clarification of Bayesian Uncertainty Convergence

Because the confidence estimation relies on variational inference with Monte Carlo dropout, additional detail is needed on how convergence is assessed. Reporting the evolution of the evidence lower bound (ELBO), validation loss, and predictive-variance distribution over training epochs would clarify when the Bayesian approximation is stable. Furthermore, specifying how many stochastic forward passes are required for a reliable estimate of the mean and variance would provide practical guidance for computational efficiency.

We assessed convergence of the Bayesian approximation both at the level of the variational objective (as a proxy for ELBO) and at the level of the posterior predictive distribution. In the implementation of variational inference by MC dropout proposed by Gal & Ghahramani [1] and implemented here, MC-sampled dropout during training corresponds to minimising a stochastic variational objective consisting of the expected negative log-likelihood and a KL term approximated by L_2 regularisation. The training loss therefore provides a Monte Carlo estimate of the negative evidence lower bound (ELBO). Stabilisation of the training and validation losses therefore indicates convergence of the variational objective. As shown in Supplementary Figure S6c of the manuscript, both losses stabilise early during training, indicating that the optimisation of the variational objective has converged. To evaluate convergence of the posterior predictive distribution, we computed the voxel-wise predictive variance after each training epoch using 15 stochastic forward passes. **Figure R4a** shows the predictive variance mapped onto the EM density after selected epochs, which stabilises rapidly after the third epoch. This is also evident from the corresponding variance distributions shown in **Figure R4b-c**. The distributions are consistently right-skewed, with only a small fraction of high-variance voxels, and the bulk of the distribution, particularly around the

mean, stabilises after approximately the third epoch. This early stabilisation indicates that the expected predictive variance has converged and supports its use as a reliable calibration target for predicting average residuals.

Regarding the number of stochastic forward passes required for inference, Supplementary Figure S6g in the manuscript evaluates the rate of change of the predicted mean and variance as a function of the number of stochastic forward passes. Both quantities converge after approximately 15 forward passes. This value is therefore used as the default during inference in LocScale-FEM, providing a practical compromise between computational efficiency and stability of the uncertainty estimates.

[1] Y. Gal and Z. Ghahramani. Dropout as a Bayesian Approximation: Representing Model Uncertainty in Deep Learning. In Proceedings of the 33rd International Conference on Machine Learning, pages 1050–1059. PMLR, [2016]

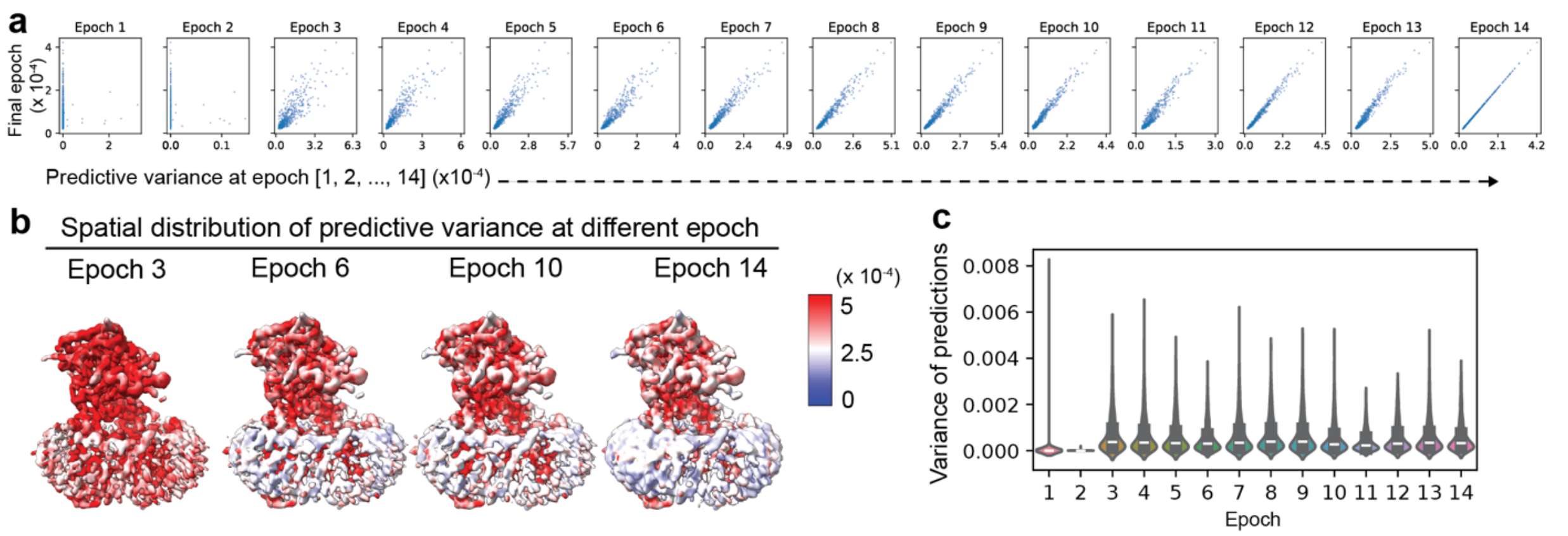

Figure R4 | Convergence of predictive uncertainty during variational training. (a) Scatter plots comparing voxel-wise predictive variance estimated at different training epochs with the variance obtained after the final epoch (epoch 15). Each panel shows 5,000 randomly sampled voxels. Rapid convergence toward the final variance estimates is observed after the early epochs. (b) Spatial distribution of predictive variance mapped onto the EM density at selected. Variance patterns stabilise early during training. (c) Distribution of predictive variance across epochs shown as violin plots. The distributions remain consistently right-skewed with only a small fraction of high-variance voxels, while the bulk of the distribution stabilises after approximately the third epoch, indicating convergence of the posterior predictive variance.

Output Deliverables

It is currently unclear what specific data products are produced by LocScale-2.0. The manuscript should explicitly list the output files and their intended usage. For example:

- *The sharpened or locally scaled map (MRC/CCP4)*
- *The voxel-wise confidence or uncertainty map*
- *Associated metadata (e.g., local B-factor volume, masks, run parameters, and log files)*
- *Are the dummy atom files available?*

Including a schematic of the output hierarchy would clarify which maps should be deposited in EMDB versus used internally for model building.

We thank the reviewer for this suggestion. LocScale-2.0 retains all intermediate files and exposes these to the user at two directory levels. The main folder contains the final optimised maps and the auxiliary files required for their interpretation, while all intermediate files are stored in the processing files folder. For the physics-informed optimisation workflow (Hybrid LocScale), the main output is the optimised LocScale map. For LocScale-FEM, the main outputs are the feature-enhanced map, the baseline map, the MRC file containing the pVDDT confidence scores, and a ChimeraX script that can be launched for immediate visualisation of the feature-enhanced map with the pVDDT confidence scores mapped onto the density. To clarify the output structure, we have included an additional supplementary figure (shown below) that lists the most relevant output files generated by both workflows.

We also thank the reviewer for raising the important question of EMDB deposition. This is not entirely straightforward, as the choice of the primary map has implications for the EMDB validation workflows. For example, the primary map is used

for map-model validation and reporting of the associated statistics. At present, however, there is no universally adopted standard defining what constitutes the primary map (e.g., minimally processed maps versus (deep-learning) post-processed maps). This lack of standardisation is unfortunate and complicates the choice of which map should be deposited as the primary map.

In view of current EMDB deposition practices, our practical recommendation is that, along with the mandatory unfiltered half-maps, the LocScale or LocScale-FEM optimised map should be deposited as the primary map if it served as the main basis for model building and figure generation, while the baseline map should be deposited as an additional map. For LocScale-FEM maps, we further consider it essential that pVDDT confidence maps are deposited alongside the optimised and baseline maps, as these provide important information for interpreting the reliability of enhanced features. Although the MRC files containing the pVDDT confidence scores are not physical density maps but voxel arrays in which each voxel stores the associated confidence score (similar in concept to local resolution maps), we have confirmed with the EMDB staff that deposition of such auxiliary volumes will be possible and are currently discussing appropriate metadata requirements (Kyle Morris, personal communication).

More generally, however, we believe that the question of what constitutes the most appropriate primary map for deposition and validation would benefit from broader discussion within the cryo-EM community and with the EMDB to establish clear recommendations.

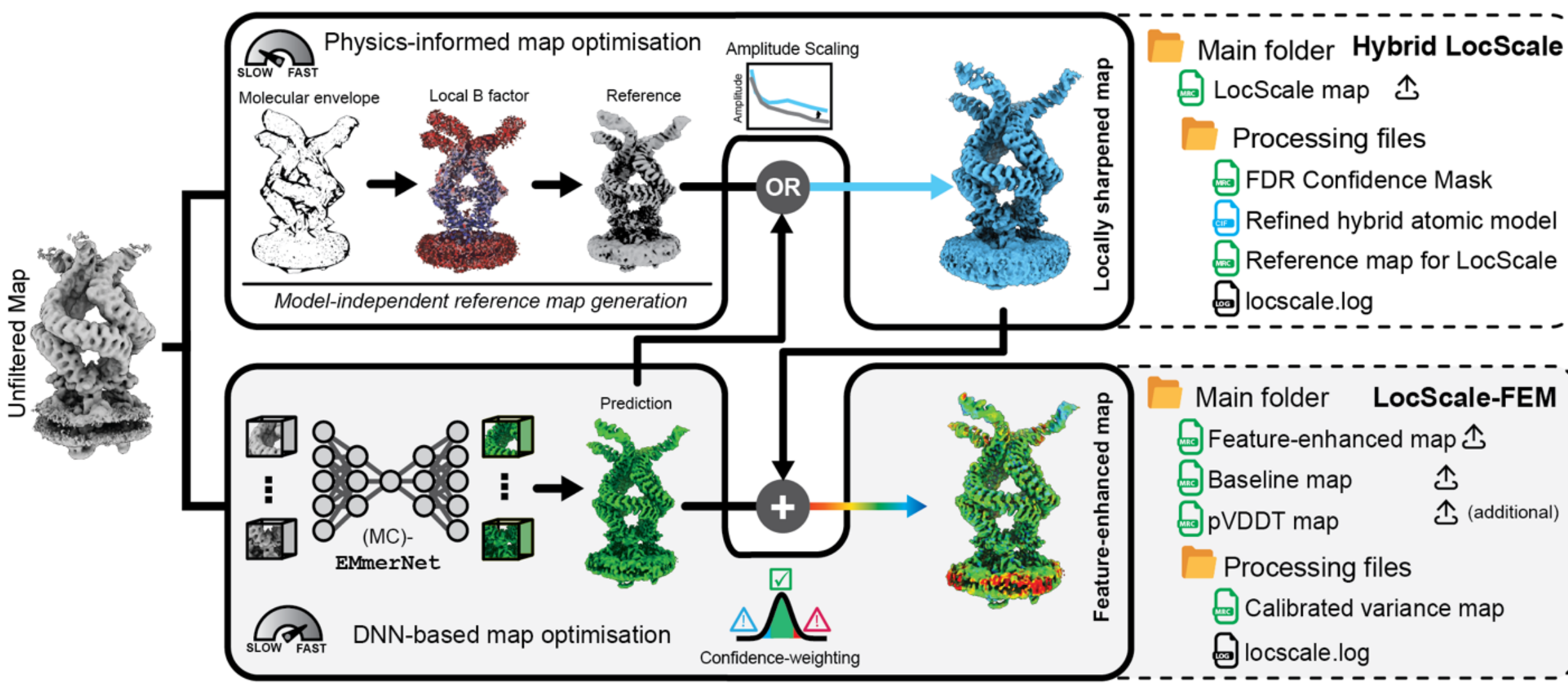

Output structure of LocScale-2.0 | Overview of the main output files generated by the Hybrid LocScale and LocScale-FEM workflows, including final optimised maps and intermediate processing files used for map optimisation and uncertainty estimation.

Comparison to Other Density Modification Approaches

The authors compare LocScale-2.0 primarily to Phenix.autosharpen, yet omit direct comparison to Phenix.resolve_cryo_em (formerly resolve_em), which, unlike autosharpen, is also a density modification algorithm that incorporates local map–model priors and solvent flattening. Given that resolve_em and LocScale-2.0 share conceptual similarities in their treatment of local density weighting and prior-informed amplitude optimization, it would be important to understand why this comparison was not performed.

The authors should clarify whether such analyses were attempted, and if not, provide justification (e.g., differing input requirements, parameter incompatibilities, or computational limitations). Ideally, inclusion of one or two head-to-head comparisons—using identical half-maps and masks—would contextualize the claimed improvements relative to other modern density-modification methods and help delineate the specific advantages conferred by the Bayesian confidence framework introduced here.

We thank the reviewer for this suggestion. In Figures 3 and S4a we compare LocScale-2.0 (Hybrid LocScale) to phenix.autosharpen because both methods are local sharpening methods, i.e. they adjust the relative scaling of amplitudes of the Fourier coefficients in the Fourier series representing the target map while leaving the experimental phases unchanged. In Figures 6-7 and Supplementary Figures S7-S8 we compare LocScale-FEM to DeepEMhancer and EMReady, because all three methods use learned models to predict optimised maps directly from the input density. Phenix.resolve_cryo_em, by contrast, is a density modification method. It differs from the above approaches in that it performs iterative real-space density modification steps combined with reciprocal-space updates of amplitudes and phases. While the hybrid scaling procedure in LocScale-2.0 also uses priors in local density weighting, it does so only for the amplitude but not the phase component.

Nevertheless, we agree that a direct comparison is informative. As per the reviewer's suggestion, we performed head-to-head comparisons for three of the examples used in the manuscript using identical half-maps and masks as inputs. The results are shown in **Figure R5**. The results confirm the observations in the manuscript that LocScale-2.0 performs similarly in enhancing contrast and detail in well-resolved regions and broadly outperforms the other methods in simultaneously preserving weak contextual density in the optimised maps.

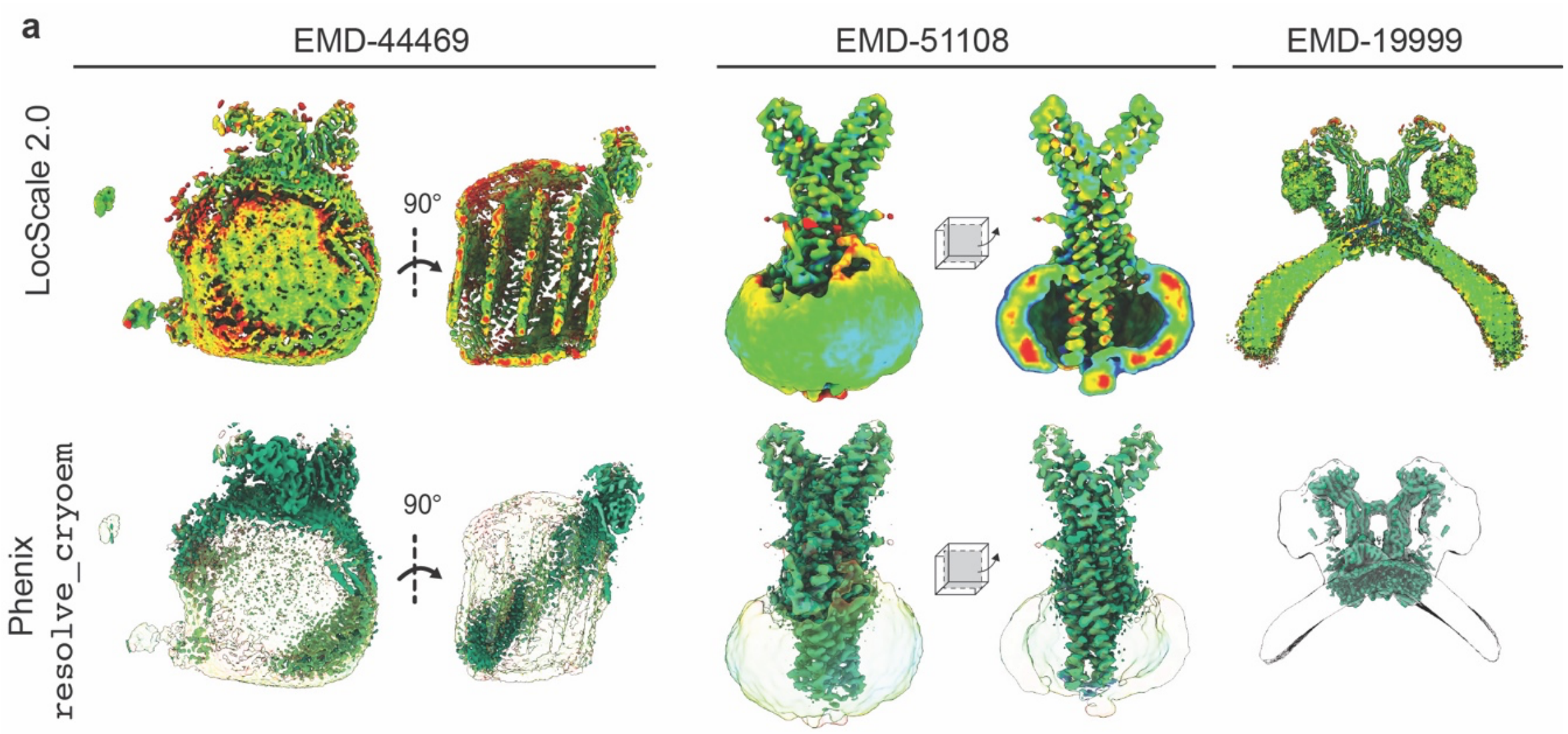

Figure R5 | (a) Comparison of LocScale-2.0 map optimisation with phenix_resolve_cryo_em. For the phenix.resolve_cryo_em maps the outline of the FDR-estimated molecular boundary is shown in transparent colour. For the tested examples LocScale optimises both high-resolution detail/contrast and of contextual density, whereas contextual density is almost completely suppressed in the phenix_resolve_cryo_em maps.

Evaluation of Heterogeneity versus Local Resolution

One of the most promising potential uses of LocScale-2.0 lies in its ability to differentiate between regions of intrinsic structural heterogeneity and those of reduced local resolution arising from imaging or alignment limitations. The authors should explore this explicitly using test cases known to exhibit conformational variability, such as spliceosome assemblies, ribosomal intermediates, or viral capsid expansion states where compositional and conformational heterogeneity are well characterized in the literature.

Applying LocScale-2.0 to such systems could demonstrate whether voxel-wise confidence values correlate more strongly with true conformational variability (e.g., multiple states or flexible domains) rather than simply local resolution estimates. This would not only validate the interpretability of the uncertainty metric but also help establish the method's value as a diagnostic tool for distinguishing dynamic from static disorder within complex macromolecular machines.

We thank the reviewer for this suggestion and for highlighting an important and active area of cryo-EM methodology. We would like to clarify, however, that LocScale-2.0 is not designed to distinguish between intrinsic structural heterogeneity and reductions in local resolution arising from imaging, alignment, or reconstruction limitations, nor do we make such a claim in the manuscript.

From the perspective of the reconstructed density map, both intrinsic conformational heterogeneity and reduced local resolution manifest as local blurring or attenuation of high-frequency signal. LocScale-2.0 operates on the map representation itself and therefore cannot disambiguate the underlying cause of this signal loss. Regions affected by conformational variability, compositional heterogeneity, or alignment-related resolution anisotropy are treated equivalently, as all lead to locally reduced signal contrast in the reconstruction. As such, while LocScale-2.0 can adaptively rescale map amplitudes based on local signal properties, it does not attempt to infer whether these properties arise from physical heterogeneity or from limitations of the reconstruction process. Addressing this distinction typically requires complementary approaches, such as focused classification, or heterogeneous reconstruction methods, which operate upstream of map optimisation.

Minor Comments

• Clarify whether reported confidence values are absolute or relative within each reconstruction; this distinction is critical for inter-map comparison.

As described in the main text and methods, the reported pVDDT confidence metric is absolute within and across reconstruction. A particular confidence score therefore has the same meaning for every density map obtained using the LocScale-FEM method.

• Ensure consistent terminology throughout: if "confidence-guided map" and "uncertainty-weighted map" refer to the same output, state this explicitly.

We now use consistent terminology (confidence-guided) throughout.

• Including example ChimeraX session files or command-line scripts would enhance reproducibility.

For the reviewers convenience, we have made all ChimeraX session files used for generation of figures in the manuscript available at https://surfdrive.surf.nl/s/MW8Bfdb3HD3P9Rq. All other scripts and relevant data to reproduce plots in the manuscript are available from https://gitlab.tudelft.nl/aj-lab/publications.

Summary

LocScale-2.0 represents an important and technically ambitious evolution of local scaling methods, incorporating Bayesian confidence estimation into cryoEM map refinement.

We thank the reviewer for their encouraging comment.

Reviewer #2:

The manuscript by Bharadwaj et al. represents a significant improvement over their successful pioneer work in map enhancement, LocScale (1.0). The original work had a key limitation in that it required substantial prior knowledge of the structure to be enhanced (or a closely related one), a shortcoming that has been addressed in this new "version." Additionally, the authors introduce a deep learning "version" of their method, which offers substantial speed, enabling them to provide some form of confidence limits.

The manuscript is well written despite the large amount of information presented. I still suggest some ways to further improve it.

We thank the reviewer for their encouraging comments and their suggestions.

The main body of the manuscript could be more schematic while conveying some key information more clearly. In particular, in the main text it is indicated that instead of the prior information demanded by their older method, now they use "First-principle expectations and data-driven approaches"… which, as such, is not very informative for the average user (in the Methods section, the description is very clear, with REFMAC as a central approach). Could the authors present a bit more in the central part of the work, what these "first principles" are?

We thank the reviewer for this suggestion. We agree that the description of the "first-principle expectations" used in the optimisation procedure can be made clearer in the main text. In the revised manuscript, we have therefore reworked the

relevant section to introduce the core concepts earlier. In particular, we now briefly outline the physical assumptions underlying the procedure, namely the expected behaviour of radially averaged structure factors of biological macromolecules and their dependence on mass distribution, local B-factors, and secondary structure, before referring to the detailed methodological implementation described in the Methods section.

On the Figures, in general, they are so loaded with data, and the labels are so small, that in the printed copy of the manuscript many labels are unreadable.

We thank the reviewer for pointing this out. We acknowledge that several figures contain a substantial amount of information, as they are intended to illustrate the behaviour of the method across multiple representative examples. At the same time, other reviewers requested additional data to further demonstrate robustness, which limits how much material can be removed.

Nevertheless, we have revised the figures to improve readability where possible. In particular, we have increased label sizes, simplified annotations, and reduced visual clutter where feasible while retaining the key information necessary to support the conclusions.

Then, in the deep learning section, the motivation is clear, and the authors explain their calibration approach well. However, since the confidence measure comes from repeatedly applying the same method, I wonder if they might have a systematic error or bias that calibration cannot fix.

We agree that Monte Carlo dropout samples are generated from the same trained network and may therefore share systematic biases that calibration alone cannot remove. Calibration corrects the scale of predicted uncertainty (i.e., over- or underconfidence relative to observed residuals), but it does not eliminate bias in the mean prediction.

To mitigate this limitation, our confidence metric is not based solely on dropout variance. Instead, pVDDT is computed by statistically comparing the feature-enhanced prediction to a phase-conservative baseline map derived from the experimental half maps. Because this baseline retains the experimental phases, it provides a reference that is independent of the learned phase modifications introduced by the network. Systematic artefacts in the network prediction therefore appear as statistically significant deviations relative to this baseline and are explicitly flagged by the pVDDT score. In addition, in response to a question from Reviewer 1 we introduced a cross-FSC analysis (**Figure R3**) that evaluates half-map derived LocScale-FEM maps against the independent opposite half-map and thereby also provides a sensistive test to putative bias. Our results indicate that this is not the case. We therefore consider this analysis a useful complementary validation to be incorporated as a routine diagnostic in the LocScale-FEM workflow.

Out-of-distribution errors, limitation of training data: In the method section, I do not understand the relationship with DeepEMhancer (why does the training set correspond to the one used by that method?). Much has changed since DeepEMhancer was introduced/trained.

We thank the reviewer for raising this point and apologise for the lack of clarity. DeepEMhancer was trained on maps optimised with LocScale-1.0, i.e. using atomic model–based local sharpening. By reusing the same underlying dataset of EMDB-deposited cryo-EM maps, but regenerating the training targets with the hybrid, model-independent LocScale-2.0 procedure, we enable a direct (and fair) comparison between the two optimisation strategies. In this way and for the purpose of this paper, the observed differences in DeepEMhancer-modified maps and the LocScale-FEM maps reflect the map optimisation framework rather than differences in dataset composition.

We nevertheless certainly agree with the reviewer that the EMDB has expanded substantially since the introduction of DeepEMhancer, allowing training on a more sequence- and structure-diverse set of cryo-EM maps. We have re-trained LocScale-FEM using such expanded datasets and provide comparative results below, which shows improvements even beyond those reported in the manuscript (**Figure R6**). Nevertheless, for the central methodological comparison presented in the paper, we believe that the strategy outlined above offers the fairest and most informative basis for evaluating differences between the different map optimisation approaches.

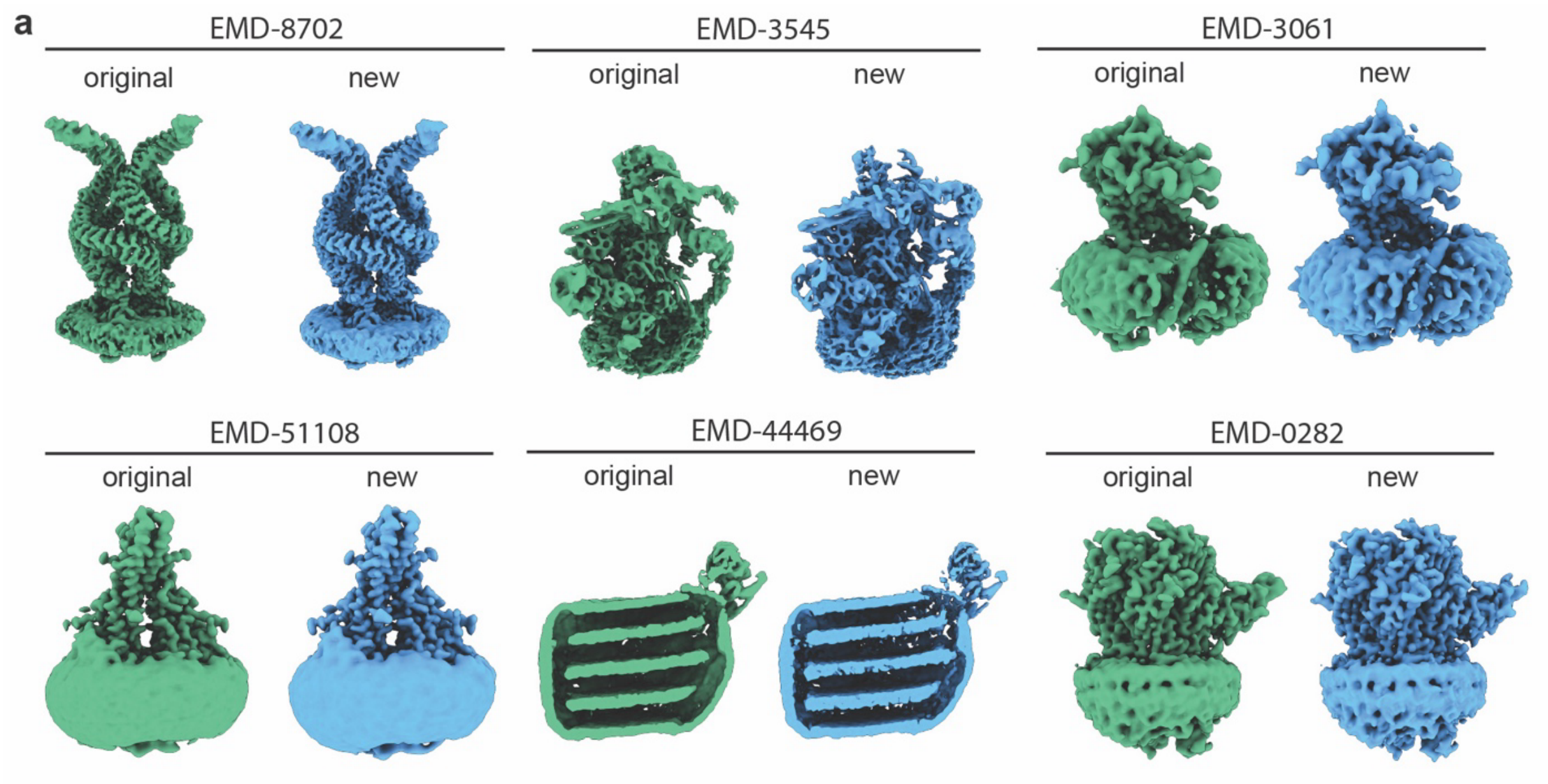

Figure R6 | (a) Comparison of LocScale-FEM map optimisation using the original model and a new model trained on an expanded training set. The expanded model preserves contextual density similarly to the original LocScale-FEM model while showing local improvements in contrast in well-resolved, high-resolution regions.

Finally, although I understand that the method works specifically in Fourier space and not in real space, I suggest that popular quality measures like Q-factor could also be included, perhaps as Supplementary Material.

We thank the reviewer for the suggestion. As explained in our response to Reviewer 1, we consider the use of Q-scores inappropriate in this context because they constitute a form of circular validation. Q-scores measure how well the experimental maps fit with Gaussian-like functions around atoms and therefore are inherently biased in methods trained on target maps directly or indirectly derived from a Gaussian forward model. LocScale-2.0, like DeepEMhancer and EMReady, would therefore be expected to show improved Q-scores; however, such improvements reflect a training "artefact" rather than an independent measure of map quality. We think a better and more objective way of true measuring map improvement is the average cross-FSC strategy outlined in Figure R3 and now part of the revised manuscript in Figure S7g-h.

Reviewer #3:

The authors present an AI method to modify the density of a cryoEM reconstruction. The method relies on a previous physics-based method to adjust the amplitudes of cryoEM maps to better represent the included mass of the particle. It makes use of filling the estimated volume with pseudo-atoms, thus not relying on a specific model. However, as the authors state, the pseudo-atomic approach is useful at lower spatial frequencies and does not capture the high resolution details. To extend resolution, the method still relies on specific models, and may still retain some bias. Additionally, the method relies strongly on adequate calibration and numerous adjustments, which depends on the parameters the user chooses. This drastically increases the likelihood of generating artifacts, as well as complicating interpretation. The main problem is that it depends on a series of interpretations, where introducing some bias in one step propagates and magnifies in the next. Therefore, in careful hands, this method could be used to generate details in maps that could be subsequently tested by other methods. The possibility of generating artifacts should be addressed by the authors where they discuss the limitations. With these issues raised, I believe it is a useful study that warrants publication.

We thank the reviewer for their evaluation and for acknowledging the potential utility of the approach. We would like to clarify several points where our intent and implementation appear to have been misunderstood.

First, a central design goal of LocScale-2.0 and LocScale-FEM is precisely to remove user-tunable parameters from the map optimisation workflow. The physics-informed LocScale-2.0 procedure estimates all required quantities automatically

from the data or from empirical considerations explained and derived in the manuscript, and the deep-learning component (LocScale-FEM) is trained to emulate this fully automated workflow. As such, irrespective whether the physics-based or deep-learning based routes of LocScale-2.0 are chosen, the method does not rely on user-selected parameters or manual calibration steps, and we explicitly avoid exposing additional degrees of freedom that could amplify bias. The robustness of this automated behaviour is illustrated in Figures 2–3 and Supplementary Figures S1–S4. We also invite the reviewer to run the program themselves with default parameters.

Second, the hybrid LocScale-2.0 procedure operates exclusively on Fourier amplitudes and therefore has a very limited capacity to introduce phase bias. This design choice, and its implications for bias propagation, are discussed in detail in in our earlier work (Jakobi et al., PMID 29058676; Bharadwaj & Jakobi, PMID 35938593). Because no phase information is imposed or modified during the hybrid LocScale-2.0 optimisation, the method is restricted to spectral reweighting existing signal rather than introducing new structural features. While the deep-learning-based LocScale-FEM workflow does not share this strict phase constraint, the approach incorporates additional safeguards to mitigate the introduction or propagation of bias, which is the central motivation for the uncertainty-aware framework presented in our manuscript.

Third, we agree that any learned post-processing method carries a risk of generating artefacts if applied outside its regime of validity. Addressing this concern has been a primary motivation for introducing the pVDDT confidence score (section "Feature-enhanced maps from uncertainty-aware deep learning" in the original manuscript). The pVDDT score quantifies the statistical confidence of prediction-induced deviations relative to a phase-conservative baseline derived from the experimental half maps. Regions where the network prediction deviates strongly from this baseline are therefore explicitly flagged, and we emphasise throughout the manuscript that feature-enhanced maps should always be interpreted together with their associated confidence scores.

Finally, we note that the method is not intended to *generate* structural detail but rather to enhance the interpretability of existing signal while highlighting regions where the reliability of predicted features is uncertain. We believe these safeguards and limitations are discussed comprehensively in the manuscript, and we have further clarified this point in the revised Discussion to ensure that the intended scope and appropriate use of the method are clear.

I find the results very complicated to interpret and was not able to properly comment on the details. I therefore focused on the methods:
Line 669: The density is in g/cm3 and Avogadro's constant is in #/mol, but the volume is presumably in Å3 and the average atomic mass is in Da. It would be better to have relatable units, such as Da/Å3 for density.

We report protein density in g/cm^3 following common convention in the biophysical community but agree relatable units are desirable. For clarity, we additionally provide the equivalent value in Da/Å^3 in the revised text.

Line 744: I don't understand the relevance of REFMAC here. Please clarify.

REFMAC is used here exclusively for restrained isotropic B-factor (ADP) refinement of the pseudoatomic, atomic, or hybrid reference models against the unmodified cryo-EM map. The refined local B-factors provide a physically motivated estimate of local signal decay that is subsequently used to construct reference structure factors for local amplitude scaling in LocScale-2.0.

Line 814:
Where did these "LocScale-2.0 reference maps" come from? Were they calculated from existing models?

The reference maps are generated by applying the physics-based LocScale-2.0 procedure.

Line 847:
What does dropout mean? How does it compare with the Metropolis criterion?
Dropout refers to the random omission of network units (or weights) during each forward pass, where units are independently set to zero with a fixed probability. While originally introduced as a regularisation technique, dropout admits a probabilistic interpretation: each realisation of the dropout mask corresponds to a sample from a variational distribution over the network parameters. By retaining dropout at inference and performing multiple stochastic forward passes (Monte Carlo dropout), we obtain samples from an approximate posterior predictive distribution, which constitutes a form of

variational Bayesian inference (see Gal and Ghahramani, PMLR 2016). This approach differs fundamentally from Markov Chain Monte Carlo methods, which rely on proposal mechanisms and acceptance–rejection steps such as the Metropolis criterion. Monte Carlo dropout does not construct a Markov chain and does not require an acceptance criterion; instead, uncertainty is captured directly through repeated sampling from the variational approximation induced by dropout.

Reviewer #4:

The manuscript presents LocScale-2.0, a map-optimization framework aimed at handling spatially heterogeneous cryo-EM reconstructions by enhancing local detail without requiring prior structural knowledge. The approach combines (i) a physics-informed, model-independent procedure that generates local references and performs local Fourier-amplitude scaling, and (ii) a deep-learning approach (MC-EMmerNet) trained to emulate the physics-informed output and to produce feature-enhanced maps (LocScale-FEM) alongside voxel-wise confidence scores (pVDDT).

Overall, I find the contribution potentially high-impact and practically useful, especially for reconstructions with contextual density (e.g. membranes, glycans) where common sharpening/denoising may suppress biologically relevant features. It appears to improve on DeepEMhancer, especially by providing a confidence-guided deep-learning post-processing framework. The manuscript is also clearly written. However, in my view the manuscript cannot be accepted before major revision, because the current training/evaluation setup does not yet define a sufficiently realistic scope of validity for the deep-learning component nor demonstrate robust generalizability of the confidence estimates.

We thank the reviewer for the encouraging comments.

Major concerns:

1) Scope and realism of the data augmentation strategy

The augmentation strategy (random rotations; random sharpening/blurring using B-factors 0–400 Å^2; Gaussian low-pass filtering) mainly introduces isotropic, smoothly varying spectral changes. In contrast, loss of high-resolution information in experimental cryo-EM maps is often anisotropic, spatially heterogeneous, and non-Gaussian, driven by residual CTF effects, preferred orientation, beam-induced motion, masking/reconstruction artifacts, and locally weak/partially ordered components.

While the chosen augmentations are described clearly, the manuscript does not evaluate whether they capture these experimentally relevant distortions. As a result, the network may be optimized for patterns consistent with the LocScale-2.0 reference maps, while its behavior on maps dominated by anisotropy and locally heterogeneous signal remains insufficiently characterized. In such cases, the physics-informed, phase-conservative workflow may be the safer option. Consequently, a more systematic evaluation of the method's correctness on maps exhibiting pronounced anisotropy and locally heterogeneous signal would be necessary to delineate its range of validity and reliability.

We thank the reviewer for raising this point. The purpose of the augmentation scheme is not to reproduce the full range of experimental distortions that may occur in cryo-EM reconstructions, but rather to improve the robustness of the network when learning to emulate the physics-informed LocScale-2.0 optimisation procedure. The applied augmentations introduce variations in spectral behaviour and signal strength that are sufficient for the network to learn the mapping between unprocessed maps and LocScale-2.0 target maps form physics-informed optimisation.

The method's robustness to map anisotropy is discussed further in our response to Reviewer 1. The reviewer also mentions locally heterogeneous contrast loss and other experimental effects such as beam-induced motion or partly ordered components. We note that all these effects are absorbed by the (local or global) B-factor of the reconstruction and thus are inherently captured in our model. Importantly, such effects are already present in the deposited EMDB maps used for training. Since the LocScale-2.0 optimisation procedure is applied to these maps to generate the training targets, the network effectively learns to reproduce how the physics-informed method handles these effects. Certain artefacts, such as masking artefacts introduced during refinement or reconstruction, cannot be corrected by post-processing methods. However, the network operates on unfiltered half maps, which in principle should not be subject to masking or filtering. Importantly, the physics-informed LocScale-2.0 workflow itself does not rely on these augmentations and therefore always serves as a reference procedure; we also emphasise that a phase-conservative baseline map is a default output also as part of the LocScale-FEM workflow.

2) Size, diversity, and characterization of the training and evaluation datasets (incl. ligands)

The authors are commended for listing all EMDB/PDB entries used. However, the deep model is trained on 114 EMDB/PDB entries, which is a limited number of independent maps. Although overlapping 32 Å sub-volumes substantially increase the number of training samples, many patches originate from the same maps, reducing the effective diversity of the dataset. The manuscript also does not report structural redundancy (e.g., homologs, repeated architectures, similar complexes in different states), which could inflate apparent generalization.

We thank the reviewer for raising this point regarding the size and diversity of the training dataset. First, we note that in response to Reviewer 1, we trained an additional LocScale-FEM model using a substantially expanded training dataset. The results of this experiment are shown in **Figure R6**, where the expanded model produces results very similar to those obtained with the original model, while showing minor improvements in local contrast for well-resolved regions. This indicates that the performance of the method is not strongly limited by the size of the original dataset.

With respect to structural redundancy, we have added the following to the Methods section: "*Redundancy control was applied following the same procedure as outlined in PMID 34267316. EMDB entries were grouped according to 30% sequence of identity clusters, and the training, validation, and test sets were constructed such that they are mutually exclusive under this criterion. To avoid bias during evaluation, only one representative per sequence cluster was included in the validation and test sets.*"

The uncertainty calibration test set comprises 11 maps, which may limit robustness across heterogeneous targets. In addition, the manuscript would benefit from a statistical summary of dataset properties (resolution distribution, modality/instrumentation, biological classes), and stratified performance across resolution ranges; currently quantitative analysis focuses largely on ~3–6 Å leaving performance at lower resolutions insufficiently characterized. A clearer assessment of dataset diversity and redundancy, together with stratified evaluations across resolutions and structural classes, would help delineate the range of applicability and generalizability of the methods.

We thank the reviewer for raising the issue of the diversity of the calibration test set. If the calibration set were not representative, this would indeed limit the utility of pVDDT scores. To test this, we performed calibration once again with a completely new dataset and checked for consistency with the original calibration model.

To test this, we repeated the calibration procedure using a completely independent dataset and compared the resulting calibration model with the original one. Specifically, we randomly selected 50 EMDB entries spanning a broad range of reported resolutions (Figure R7a). We ran the standard LocScale-FEM workflow to obtain the original pVDDT scores and then recalibrated the predictive variance with the new calibration set following the procedure described in the Methods section (**Figure R7b-c**), producing a new calibration model. We then compared pVDDT scores computed using this new model with those obtained from the original calibration model. This comparison was performed on four additional EMDB entries at different resolutions and from diverse structural classes that were not included in either calibration dataset.

As shown in **Figure R7d**, the resulting pVDDT scores remain broadly consistent regardless of the calibration model. This consistency is visualised using scatter plots of the original and recalibrated pVDDT scores for 5000 randomly sampled voxels within the calibration range. The strong agreement between the two sets of scores indicates that the uncertainty calibration is robust to the choice of calibration dataset, supporting its use as a guide for map interpretation. The reviewer also raises an interesting point regarding performance across different resolution ranges. While the uncertainty calibration itself remains stable across a broad resolution range (**Figure R7d**), we observe that at lower resolutions the distribution of pVDDT scores shifts toward more positive values, indicating that the network tends to increase intensities relative to the phase-conservative baseline. This behaviour is consistent with the intended objective of a map optimisation tool, as lower-resolution maps typically exhibit attenuated density that benefits from enhanced contrast.

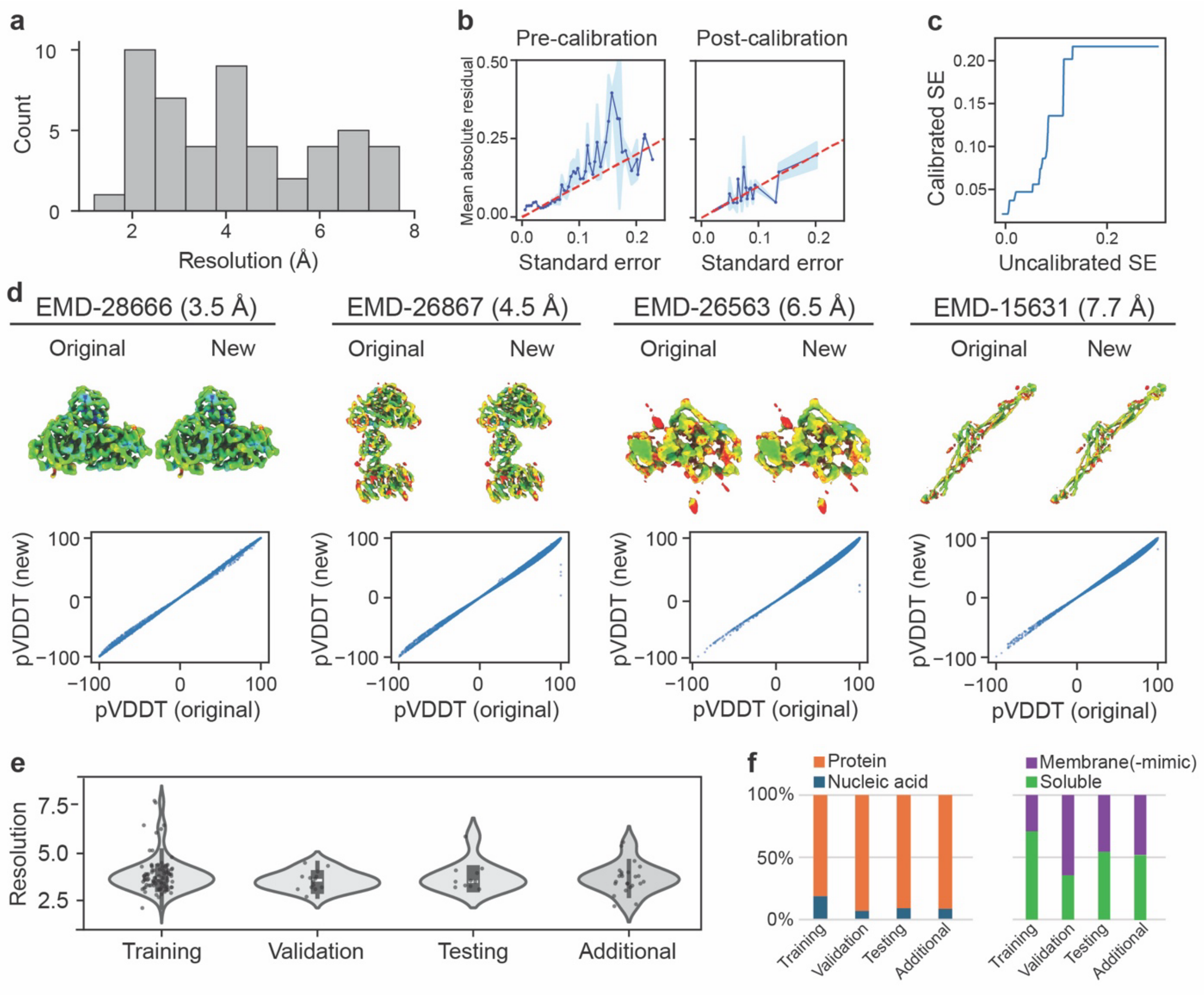

Figure R7 | **Robustness of pVDDT calibration and dataset statistics**. (a) The distribution of reported resolution for all the EMDB IDs in the new calibration dataset (N=49). (b) Reliability curve used to evaluate the uncertainty calibration (b) Isotonic regression model used for uncertainty calibration (d) pVDDT scores from the original and re-calibrated model mapped to LocScale-FEM density maps of representative structure from four different resolution bins (EMDB: 28666, 3.5 Å; EMDB: 26867, 4.5 Å; EMDB: 26563, 6.5 Å; EMDB: 15631, 7.7 Å). Scatter plots comparing the pVDDT scores at a random sub-sample of 5000 voxels within the calibration range (mean predicted intensity > 0.1) using the original and new calibration dataset are shown below each representative map pair. (e) The distribution of reported resolution for all the EMDB IDs used in the training, validation, test and additional sets used in this paper. (f) Statistical summary of biological classes of EMDB IDs in different datasets

The reviewer requested a statistical summary of dataset properties, and we provide the relevant information in **Figure R7 e-f**. LocScale-FEM EMmerNet model was trained using maps spanning a broad range of input resolutions and structural classes (see Figure R7e and Methods), and the validation, testing, and additional datasets used in this study exhibit statistically similar resolution distributions. In **Figure R7f** we also provide a summary of relevant biological classes to which the different EMDB IDs belong. Overall, 15% of the EMDB IDs contain nucleic acids, and their share among the various subsets varies between 8% and 20%. Further, we also report the percentage of entries containing membrane or membrane mimics in different datasets. 33% of all structures contain membrane or membrane-mimic density and their fractions vary between 30% and 64% among different subsets.

While datasets may differ in (optical) characteristics of microscopes, detectors used and imaging conditions, most of these parameters manifest primarily in the dampening envelope and are therefore effectively captured through local B-factors (see Jakobi et al., PMID 29058676 and Bharadwaj & Jakobi, PMID 35938593). We therefore believe that reporting a detailed summary of the instruments and imaging conditions used for the different datasets would add limited additional insight for the present analysis and have not included this information in the figure.

3) Ligands: I appreciate that ligands are considered, but I'm concerned about whether there is sufficient training diversity. A small number of maps can provide extensive protein-density examples, but ligands are a small fraction of voxels/models, so generalisation may be weaker precisely where over-interpretation risk is highest.

We thank the reviewer for this point. As discussed in the manuscript, the pVDDT confidence score measures the confidence in the prediction of the feature-enhanced map relative to a phase-conservative baseline map. As such, it is explicitly designed to flag regions where the network prediction may be unreliable, including cases where training diversity is limited.

As the reviewer correctly points out, ligands represent a particularly challenging use case because they span a wide chemical space and hence training diversity is almost inherently limited. This limitation is explicitly highlighted in the manuscript (Figure 7, Supplementary S8), where we show that in some cases ligand densities may be suppressed by LocScale-FEM. In the ensuing discussion, we emphasise that ligand interpretation should always be based on joint inspection of the feature-enhanced map, the baseline map, and the confidence scores, rather than on the feature-enhanced map alone. The joint use of the phase-conservative baseline map provides an excellent means to detect out-of-distribution bias in the deep-learning optimised maps of LocScale-FEM maps, and we believe this prediction + baseline/pVDDT score strategy represents a distinctive advantage of LocScale-FEM over other deep learning-based map optimisation methods where such bias would unavoidably go unnoticed.

Minor point:
I found one typo: "interpretatioo" → "interpretation".

We have corrected the typo.

Rebuttal NCOMMS-25-79735B

Reviewer #1

I thank the authors for their thorough, thoughtful, and very clearly articulated responses to all points raised. The additional analyses and clarifications substantially strengthen the manuscript, and I am fully supportive of the revised version. Looking forward to using the software in future work.

Thank you for your appreciation of our work and the encouraging comments.

Reviewer #2:

The authors have addressed all the comments we raised appropriately. They have also introduced further modifications to the manuscript, which have increased its clarity and accessibility, producing a very good and useful work for the cryoEM field. As very minor points, the authors might consider introducing the word "into RELATIVELY structural..." (Page 1, line 39)

We thank the reviewer for their encouraging comments and positive assessment. We have modified the text as suggested to clarify that, even after extensive classification, individual reconstruction classes remain averages over closely related conformations.

Reviewer #3:

The authors gave detailed responses arguing that they minimized the number of decisions users have to make. However, that means they made the decisions in code with the same possibilities of bias. I am therefore not convinced that their ML code would be immune to the typical hallucinations of AI. I would consider only the physics-based method to give reliable results. Any further extension suffers the same problems as density modification techniques taken too far. I'm therefore no comfortable with accepting the manuscript.

We respectfully disagree with the reviewer's interpretation, which appears to misunderstand a central premise of the manuscript. We do not claim that LocScale-FEM is immune to hallucinations. On the contrary, a major motivation for this work is precisely that all machine-learning-based map optimisation methods can introduce bias, particularly when used without appropriate confidence measures. A substantial part of the manuscript is therefore devoted to developing and validating a voxel-wise confidence metric designed to detect, flag, and guide interpretation of such potentially unsupported features.

We also respectfully disagree that reducing user decisions transfers user bias into code. The default choices in LocScale-2.0 are not arbitrary decisions, but are explicitly derived, parameterised, and validated in the manuscript. Automation is therefore used to make the procedure reproducible and to reduce subjective user-dependent choices, not to conceal them.

We do fully agree that the physics-informed LocScale-2.0 workflow provides the most conservative and directly interpretable optimisation route. LocScale-FEM is not presented as replacing this physics-based method, but as an accelerated, uncertainty-aware approximation whose predictions must be interpreted together with the pVDDT confidence scores. The reviewer's concern is therefore not a point we have overlooked or ignore, but rather one of the central problems the manuscript sets out to address.

Reviewer #4:

I am happy with the revision. I think the paper is ready to be published in Nat Comm.

We thank the reviewer for their supportive comment.

Reviewer #5:

The authors have address all questions and comments we had in our previous review. I consider the manuscript reday for its publication.

We thank the reviewer for their positive assessment.
